# SnRK2 subfamily I protein kinases regulate ethylene biosynthesis by phosphorylating HB transcription factors to induce *ACO1* expression in apple

**DOI:** 10.1111/nph.18040

**Published:** 2022-03-22

**Authors:** Meiru Jia, Xingliang Li, Wei Wang, Tianyu Li, Zhengrong Dai, Yating Chen, Kaikai Zhang, Haocheng Zhu, Wenwen Mao, Qianqian Feng, Liping Liu, Jiaqi Yan, Silin Zhong, Bingbing Li, Wensuo Jia

**Affiliations:** ^1^ State Key Laboratory of Agrobiotechnology China Agricultural University No.2 Yuanmingyuan West Road Beijing 100193 China; ^2^ Department of Pomology College of Horticulture China Agricultural University No.2 Yuanmingyuan West Road Beijing 100193 China; ^3^ School of Life Sciences State Key Laboratory of Agrobiotechnology Chinese University of Hong Kong EG12 Science Centre East Hong Kong 999077 China

**Keywords:** *ACO1*, ACS1, apple, ethylene, HB transcription factor, osmotic stress, SnRK2‐I

## Abstract

- Ethylene (ETH) controls climacteric fruit ripening and can be triggered by osmotic stress. However, the mechanism regulating ETH biosynthesis during fruit ripening and under osmotic stress is largely unknown in apple (*Malus domestica*).
- Here, we explored the roles of SnRK2 protein kinases in ETH biosynthesis related to fruit ripening and osmoregulation. We identified the substrates of MdSnRK2‐I using phosphorylation analysis techniques. Finally, we identified the MdSnRK2‐I‐mediated signaling pathway for ETH biosynthesis related to fruit ripening and osmoregulation.
- The activity of two MdSnRK2‐I members, MdSnRK2.4 and MdSnRK2.9, was significantly upregulated during ripening or following mannitol treatment. Overexpression of *MdSnRK2‐I* increased ETH biosynthesis under normal and osmotic conditions in apple fruit. MdSnRK2‐I phosphorylated the transcription factors MdHB1 and MdHB2 to enhance their protein stability and transcriptional activity on *MdACO1*. MdSnRK2‐I also interacted with MdACS1 and increased its protein stability through two phosphorylation sites. The increased *MdACO1* expression and MdACS1 protein stability resulted in higher ETH production in apple fruit. In addition, heterologous expression of *MdSnRK2‐I* or manipulation of *SlSnRK2‐I* expression in tomato (*Solanum lycopersicum*) fruit altered fruit ripening and ETH biosynthesis.
- We established that MdSnRK2‐I functions in fruit ripening and osmoregulation, and identified the MdSnRK2‐I‐mediated signaling pathway controlling ETH biosynthesis.

## Introduction

Ethylene (ETH) is an important hormone involved in the regulation of plant growth, fruit ripening, and stress responses (Gane, 1934; Chang, 2016; Park *et al*., 2021). Fruits are classified as climacteric and nonclimacteric based on whether or not ETH controls ripening (Adams‐Phillips *et al*., 2004). Tomato (*Solanum lycopersicum*) and apple (*Malus domestica*) are typical climacteric fruits; their ripening is controlled mainly by ETH (Giovannoni, 2004; Tatsuki, 2010; Gapper *et al*., 2013). Ethylene biosynthesis also is associated with stress (Dubois *et al*., 2018). However, the regulatory mechanisms of both ripening‐ and stress‐related ETH biosynthesis are largely unclear in climacteric fruits.

ETH biosynthesis is controlled by two types of enzymes: 1‐aminocyclopropane‐1‐carboxylic acid synthases (ACSs) and 1‐aminocyclopropane‐1‐carboxylic acid oxidases (ACOs) (Yang & Hoffman, 1984; Kende, 1993). ACSs and ACOs have been identified in many species, but it is unclear which ACO and ACS members are involved in certain biological events (Booker & DeLong, 2015; Houben & Poel, 2019). The specific *ACO* and *ACS* genes promoting ETH biosynthesis have been identified in tomato and apple fruits. *SlACS2*, *SlACS4*, *SlACS1A*, *SlACO1* and *SlACO4* are the major ETH biosynthesis genes in tomato fruits, whereas *MdACS1*, *MdACS3A* and *MdACO1* are key ETH biosynthesis genes in apple fruits (Nakatsuka *et al*., 1998; Oraguzie *et al*., 2004; Wakasa *et al*., 2006; Cara & Giovannoni, 2008; Wang *et al*., 2009).

Post‐translational regulation of ACS proteins is a major pathway controlling ETH biosynthesis, and expression levels of *ACO1* correspond well with ETH production (Pattyn *et al*., 2021). Phosphorylation mediated by mitogen‐activated protein kinase 3/6 (MAPK3/6), 14‐3‐3 proteins and target of rapamycin kinase (TOR) affect the turnover of ACS proteins, thereby regulating ETH biosynthesis in *Arabidopsis thaliana* (Joo *et al*., 2008; Han *et al*., 2010; Yoon & Kieber, 2013; Zhuo *et al*., 2020). A calcium‐dependent protein kinase (CDPK), SlCDPK2, is also involved in ETH biosynthesis; it phosphorylates SlACS2 in tomato fruits (Kamiyoshihara *et al*., 2010). However, the post‐translational regulatory mechanisms of ACS proteins in climacteric fruits remain unknown. Moreover, it is unknown whether other protein kinases are involved in ACS protein regulation. The transcriptional regulation of ACO1 has been well‐studied in apple and tomato fruits (Lin *et al*., 2008; Li *et al*., 2017). MdMYC2, MaERF11, MaMADS7, SlHB1 (LeHB‐1), SlRIN and SlNAC9 are important transcription factors that directly bind to the promoter of *ACO1* to improve its expression, thereby increasing ETH biosynthesis to accelerate fruit ripening (Ito *et al*., 2008; Lin *et al*., 2008; Xiao *et al*., 2013; J. Liu *et al*., 2015; M. Liu *et al*., 2015; Kou *et al*., 2016; Li *et al*., 2017). However, it is unclear how these transcription factors respond to ripening to drive *ACO1* expression and whether other signals or components participate in this process.

In addition to ripening, various stresses also trigger ETH production in plants (Skirycz *et al*., 2011; Zhang *et al*., 2016; Savada *et al*., 2017; Dubois *et al*., 2018). Among these stresses, drought, flooding and salt stress are closely related to osmotic stress (Skirycz *et al*., 2011; Dubois *et al*., 2018); however, it is unclear how osmotic stress triggers ETH biosynthesis (Verma *et al*., 2016; Dubois *et al*., 2018). Sucrose nonfermenting protein kinase 1‐related protein kinase 2 (SnRK2) members function in response to osmotic stress (Zhang *et al*., 2011; Fujii & Zhu, 2012; Fujita *et al*., 2013). SnRK2 is a plant‐specific family of protein kinases that has three subfamilies, SnRK2‐I, SnRK2‐II and SnRK2‐III (Hrabak *et al*., 2003; Fujii & Zhu, 2012). Previous studies of SnRK2 focused mainly on the regulatory mechanisms of SnRK2‐III members in abscisic acid (ABA)‐related biological events and osmotic responses (Zhang *et al*., 2011; Fujii & Zhu, 2012; Fujita *et al*., 2013). Recent studies showed that ABA‐unresponsive subfamily I SnRK2s (SnRK2‐I) also play important roles in osmotic stress responses by activating components of the mRNA decapping complex VARICOSE (VCS) and interacting with PA to phosphorylate two dehydrin proteins, EARLY RESPONSE TO DEHYDRATION (ERD)10 and ERD14 (Maszkowska *et al*., 2019). SnRK2 family members have been identified in apple and tomato (Sun *et al*., 2011; Chen *et al*., 2013), and SlSnRK2s have been proposed to regulate tomato fruit ripening (Sun *et al*., 2011). However, the specific roles of SnRK2 family members in both apple and tomato remain unclear. Whether and which SnRK2s are involved in fruit ripening and ETH biosynthesis, and whether SnRK2‐mediated osmotic responses are related to ETH biosynthesis require further study.

In this study, we established that MdSnRK2‐I and SlSnRK2‐I protein kinases are important regulators of fruit ripening, synergistically regulating ETH biosynthesis. During fruit ripening or under osmotic stress, the MdSnRK2‐I members MdSnRK2.4 and MdSnRK2.9 are activated and then phosphorylate the HB transcription factors MdHB1 and MdHB1 to increase *MdACO1* expression, thus enhancing ETH biosynthesis. In summary, we reveal a pathway controlling ETH biosynthesis during fruit ripening and under osmotic stress in apple fruit and demonstrate that SnRK2‐I is the key post‐translational regulator of ETH in response to ripening and osmotic stress in apple and tomato fruits.

## Materials and Methods

### Plant materials and growth conditions

Apple (*Malus* *domestica* cv Golden Delicious) were chosen for this study because their genome has been sequenced (Supporting Information Fig. S1a). Apple calli were obtained and cultured according to reported protocols (Alayón‐Luaces *et al*., 2008; Li *et al*., 2016). Apple calli were subcultured at 10‐d intervals before being subjected to gene transformation. ‘Micro Tom’ tomato plants were cultured according to reported protocols (Sun *et al*., 2011).

### Bioinformation analysis and cloning of *Sucrose nonfermenting protein kinase 1‐related protein kinase 2* (*SnRK2*) and *HB* genes

Bioinformation analysis, including phylogenetic tree construction and protein sequence alignments, was conducted as described previously (Han *et al*., 2015). To identify specific members of the *MdSnRK2* and *MdHB* gene families, the coding sequences of the *AtSnRK2* and *AtHB* genes were used as queries in a Blast search against the apple genome (http://genomics.research.iasma.it/) and NCBI database (https://www.ncbi.nlm.nih.gov/), yielding a total of nine members of the *MdSnRK2* family (designated *MdSnRK2.1‐MdSnRK2.9*) and *MdHB* family (designated *MdHB1*–*MdHB23*).

### Screening of *SnRK2* and *HB* genes involved in ETH production

The full‐length cDNAs of *SnRK2s* and *HBs* were cloned into a pCambia1304 overexpression vector and then individually transformed into *Agrobacterium tumefaciens* strain EHA105 (Lazo *et al*., 1991). The EHA105 strain was cultured as described previously (Han *et al*., 2015). The transformed calli were used to detect ETH production and gene expression.

The cloned genes were further infiltrated into tomato fruit by injection at *c.* 25 d after fruit set. The fruits were then monitored throughout development and ripening. Ten to 15 pairs of fruit were infected for each gene with the empty vector (EV) as a control. The quantitative reverse transcription (qRT)‐PCR primers are listed in Tables S3 and S4.

### Measurement of ETH production

Apple calli, apple fruit and tomato fruit samples (2 g callus or 5–8 pairs of fruit) were collected from the medium or the plants at each sampling timepoint, and were enclosed in gas‐tight containers (50 ml or 2.5 l) equipped with a sampling valve. The ETH released was determined as described previously (Li *et al*., 2015).

### Quantitative reverse transcription PCR

The qRT‐PCR was performed as described by Han *et al*. (2015). Three biological replicates were set up, and each sample (five fruit or 3 g callus combined as one sample) was analyzed at least in triplicate. The primers used are listed in Tables S3 and S4.

### Analysis of *MdSnRK2s* expression in response to different treatments

At 105 d post‐anthesis (DPA), fruit disks (10 mm diameter, 1 mm thickness) were prepared from six fruit and combined to make one disk sample (5 g) per treatment. The disc samples were first vacuum‐infiltrated for 30 min in equilibration buffer (Archbold, 1999) consisting of 50 mM MES‐Tris (pH 5.5), 10 mM MgCl_2_, 10 mM EDTA, 5 mM CaCl_2_, 200 mM mannitol and 5 mM vitamin C. Then, samples were shaken for 6 h at 25°C in equilibration buffer containing either 6% mannitol, 100 µM ABA, 200 mM NaCl or 100 µM ACC (acetyl‐CoA carboxylase). After incubation, the samples were washed with double‐distilled water, frozen immediately in liquid N_2_, and kept at −80°C until used. Each individual analysis was conducted with three replicates.

### Functional analysis of SnRK2s with stable transgenic tomato plants

The pCambia1304‐*35S*‐*MdSnRK2.4/*pCambia1304‐*35S*‐*MdSnRK2.9* constructs were transformed into *A. tumefaciens* EHA105 for stable transformation of tomato following a protocol described previously (Sun *et al*., 2006; Kimura & Sinha, 2008). To examine the effect of the heterologous and overexpression of *MdSnRK2.4/2.9* on plant growth and development, nine to 12 seedlings from four independent lines with relatively high expression of *SnRK2* were examined. For gene expression analysis, two or three fruit from each individual plant were combined as an individual sample. The primers used are listed in Tables S3 and S4.

### Virus‐induced gene silencing (VIGS) in tomato fruit

An 890‐bp fragment of the *SlSnRK2.1* and *SlSnRK2.2* genes (GenBank accession nos. AK329882 and AK327274, respectively) and a 568‐bp fragment of the *ethylene‐insensitive protein 2* (*SlEIN2)* gene (GenBank accession no. AY566238) were PCR‐amplified from tomato cDNA sources and cloned into pTRV2 to generate pTRV2‐*SlSnRK2.1*, pTRV2‐*SlSnRK2.2* and pTRV2‐*SlIEIN2*. For tomato fruit infiltration, the procedure described by Fu *et al*. (2005) was used.

### Protein extraction

Total proteins were extracted from apple calli, apple fruit and tomato fruit using the following extraction buffer (pH 7.5): 100 mM Tris–HCl, 100 mM KCl, 10% glycerol, 2 mM DTT, 1 mM phenylmethylsulfonyl fluoride (PMSF), 1% Triton X‐100 and 1× protease inhibitor cocktail. Protein concentration was measured using a BCA Protein Assay Kit (ComWin Biotech, Beijing, China) and adjusted to the same level for each sample.

### Antibody production

MdSnRK2.4‐ and MdSnRK2.9‐specific antibody against synthetic peptides (MKKIMGVHYKIPDH and PDYVHISQDCRHLL, respectively), and MdSnRK2 and SlSnRK2 common peptide (GRFSEDEARYFFQQ) were produced by Abmart (Shanghai, China). MdHB1/2 and LeHB1‐specific synthetic peptide (SEEDDGSDD) was produced by Beijing Huada. The specificity of the antibodies was tested using fruit total proteins.

### Yeast‐two‐hybrid assays

Yeast two‐hybrid (Y2H) assays were performed using the Matchmaker GAL4‐based Two‐Hybrid System (Clontech, San Francisco, CA, USA) according to the manufacturer’s instructions. Constructs were produced by cloning *MdSnRK2.4* or *MdSnRK2.9* into the pGBKT7 vector and *MdHB1* or *MdHB2* into pGADT7. The primers used for the Y2H assays are provided in Tables S3 and S4.

### Bimolecular fluorescence complementation assays

Bimolecular fluorescence complementation (BiFC) assays were performed using tobacco (*Nicotiana tabacum*) as described previously (Han *et al*., 2015). The coding sequences of *MdSnRK2.4* and *MdSnRK2.9* were amplified and cloned into the SPYNE‐YFP^n^ vector, and those of *MdHBs* and *MdEIN2* were cloned into SPYCE‐YFP^c^ (YFP, yellow fluorescent protein). Fluorescence was examined 3 d post‐transformation using a confocal laser‐scanning microscope (Fluoview FV1000; Olympus, Tokyo, Japan). The primers used are listed in Tables S3 and S4.

### Co‐immunoprecipitation assays

For co‐immunoprecipitation (Co‐IP) assays, pMDC83:MdHB1/2‐GFP and SPYNE:FLAG‐MdSnRK2.4/2.9 were co‐transformed into apple calli, with FLAG‐MdSnRK2.4/2.9 and pMDC83‐GFP being co‐transformed as a control (GFP, green fluorescent protein). Total proteins were extracted 3 d post‐transformation. An equal amount of anti‐FLAG antibody coupled protein A + G‐Sepharose beads was added to total protein samples and detected with anti‐GFP antibody.

### Mass spectrometry assays

Total proteins extracted from apple calli were incubated with glutathione S‐transferase (GST)‐MdSnRK2.9 at 4°C for 4 h, and then the MdSnRK2.9 protein and its interacting proteins were immunoprecipitated and analyzed by LC‐MS/MS.

In order to identify putative phosphorylation sites of MdHB1 and MdHB2 by mass spectrometry, 5 µg MdHB1/2‐His and 10 µg GST‐SnRK2.4/2.9 purified protein were incubated in 30 µl protein kinase buffer (20 mM Tris–HCl, pH 7.5, 10 mM MgCl_2_, 10 mM ATP) at 30°C for 30 min. The reaction mixtures were analyzed by LC‐MS/MS.

MdSnRK2 phosphorylation sites in apple flesh at different developmental stages were analyzed. Total proteins extracted from apple flesh at 85 and 105 DPA were immunoprecipitated with anti‐MdSnRK2.4/2.9 monoclonal antibody and then analyzed by LC‐MS/MS.

### *In vitro* phosphorylation assays

ADP‐Glo Kinase Assays were performed according to the manufacturer's instructions (Promega, Fitchburg, WI, USA). Activity was assessed by measuring ATP with a luminometer (GloMax, Promega, Madison, WI, USA), and the average activity value ± SD is presented. Primers used are listed in Tables S3 and S4.

### In‐gel kinase activity assays

In‐gel kinase activity assays were performed as described previously (Liu *et al*., 2017) on apple calli grown on control medium (MS agar with 3% sucrose) or on the same medium supplemented with 6% mannitol or 100 µM ACC. Radioactivity was quantified using a Typhoon 9410 imager (Molecular Dynamics, GE Healthcare, Pittsburgh, PA, USA).

### Phos‐tag mobility shift assays

Phosphoprotein mobility‐shift assays with phos‐tag reagent (AAL‐107) were used to detect phosphorylated MdSnRK2.4/2.9, SlSnRK2, MdHB1/2 or LeHB1 protein as described previously (Liu *et al*., 2017). Apple calli or fruit were treated with or without 100 µM ACC or 6% mannitol for different durations, and total proteins were extracted. For the phosphorylation of MdHB1/2 or LeHB1, the extracted total proteins were immunoprecipitated with anti‐HB1 polyclonal antibody. For the analysis of MdSnRK2.4/2.9 phosphorylation level in apple flesh at different developmental stages, total proteins extracted from apple flesh were immunoprecipitated with anti‐MdSnRK2.4 or MdSnRK2.9 monoclonal antibody. Phosphorylated SlSnRK2 was analyzed using anti‐SnRK2 monoclonal antibody. These samples were incubated with or without calf‐intestinal alkaline phosphatase (CIP) at 37°C for 30 min, and then analyzed using 12% (w/v) SDS‐PAGE gel containing 50 μM phos‐tag and 100 μM MnCl_2_. Proteins were detected with specific antibodies.

### Transcriptional activity assays

β‐glucuronidase (GUS) activity was measured using a fluorometric GUS enzymatic assay following a protocol published previously (Jefferson, 1987). The promoter sequence of *MdACO1* (designated *ProMdACO1*) was cloned into pCambia1301 to generate the pCambia1301‐*ProMdACO1:GUS* reporter construct. The CDSs of *MdSnRK2.4/2.9*, *MdHB1/2* and *MdEIN2* were cloned to generate effector constructs. The constructs were transformed into apple calli grown at 27°C on Murashige & Skoog (MS) medium supplied with 6% mannitol or 100 µM ACC. Equal amounts of total proteins were used to evaluate the effects of different effectors on GUS activity. Each experiment was repeated three times. The primers are listed in Tables S3 and S4.

### ChIP‐qPCR assay

Chromatin immunoprecipitation (ChIP) was performed on apple calli expressing MdHB1/2‐GFP or the pMDC83‐GFP EV as described previously (Gendrel *et al*., 2005). Total proteins were extracted from each sample (3 g) and the MdHB1/2 proteins were immunoprecipitated using anti‐GFP (ab290; Abclone, Boston, MA, USA) antibody. Antibodies (NoAbs) were omitted as a negative control.

The enriched DNA fragments were analyzed by qRT‐PCR using the primers listed in Tables S3 and S4. PCR reactions were performed in triplicate for each sample, and the expression levels were normalized to that of *MdActin*.

### Electrophoretic mobility‐shift assay

Electrophoretic mobility‐shift assay (EMSA) was performed using a LightShift^®^ Chemiluminescent EMSA Kit (Thermo Scientific, Waltham, MA, USA) according to the manufacturer’s instructions. Oligonucleotide probes of *MdACO1* were synthesized and labelled with 5′‐biotin (Sangon Biotech, Shanghai, China). The probes used are listed in Tables S3 and S4.

### Cell‐free protein degradation assay

A cell‐free protein degradation assay of MdHB1/2 and MdACS1 was performed as described previously (Liu *et al*., 2017). To detect the degradation of MdHB1/2, apple calli transfected with *MdSnRK2.4/2.9*‐OE were treated with 6% mannitol or 100 µM ACC, and total proteins were extracted. Equal amounts of total proteins were incubated with equal amounts of recombinant MdHB1/2‐His protein and 10 mM ATP for the indicated period. The proteins were separated by SDS‐PAGE and detected with anti‐His antibody. For MdACS1 protein degradation, 5 µg purified GST‐MdACS1 protein was incubated with 2.5 µg purified His‐MdSnRK2.4/2.9 and 10 mM ATP for the indicated periods. The proteins were separated by SDS‐PAGE and detected with anti‐GST antibody. The relative amounts of proteins were determined by densitometry and normalized to loadings determined by Actin using ImageJ (v.1.8.0).

### Statistical analysis

Samples were analyzed in triplicate, and the data are presented as means ± SD. Data were analyzed using Student’s *t*‐tests or Tukey’s honestly significant difference (HSD) test implemented in Sas (v.8.1; SAS, Cary, NC, USA).

## Results

### MdSnRK2.4 and MdSnRK2.9 regulate ETH biosynthesis in apple fruit

In order to explore the regulatory mechanism of ETH biosynthesis in apple fruit, we first measured ETH content in developing ‘Golden Delicious’ apple fruit. Ethylene content in apple fruit followed a similar pattern to that in tomato fruit, another typical climacteric fruit (Fig. S1b; J. Liu *et al*., 2015; M. Liu *et al*., 2015). Apple fruit ETH content was relatively low during the early stages of development, started to increase after 60 DPA, increased rapidly after 105 DPA, peaked at around 130 DPA and then gradually decreased (Fig. S1a). This pattern indicates that 105 DPA is a key stage for ripening‐related ETH in ‘Golden Delicious’ fruit.

Transgenic apple fruit are hard to obtain and therefore fruit calli are used widely as a mock model to study the effects of target genes on fruit ripening‐related metabolic changes in apple fruit (Tatsuki, 2010; Dong *et al*., 2011). However, it is unclear whether ETH biosynthesis is induced in apple calli. Hence, we treated ‘Golden delicious’ calli with ETH and its biosynthesis precursor ACC at different concentrations to evaluate ETH biosynthesis in apple fruit cells (Adams & Yang, 1979). A low concentration (0.5 ppm) of exogenous ETH induced ETH biosynthesis in apple calli (Fig. S1c), indicating that apple callus cells are sensitive to ETH and can biosynthesize ETH effectively. ACC often is used to induce ETH responses (Adams & Yang, 1979; Guzmán & Ecker, 1990). We found that 5 and 100 µM ACC treatment significantly increased ETH biosynthesis in apple calli compared to a control treatment (NoACC), and the effects of ACC on NoACC production were similar to those of exogenous NoACC (Fig. S1c). Based on these results, we used ACC to induce the NoACC response in apple calli in subsequent experiments.

**Fig. 1:**
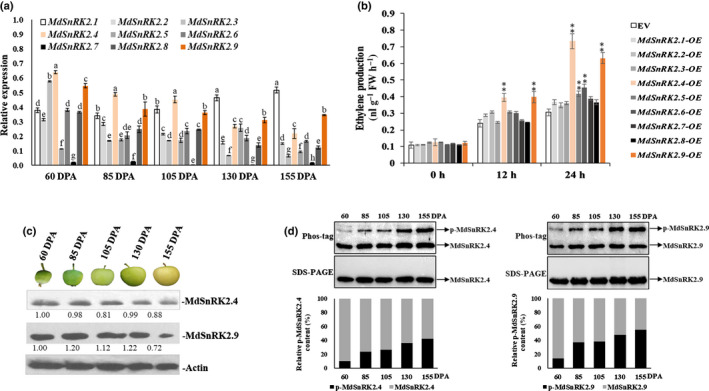
Overexpression (OE) of *sucrose non‐ferment 1 related protein kinase 2* *MdSnRK2.4* and *MdSnRK2.9* promotes ethylene (ETH) production and fruit ripening in apple [Correction after first publication 22 March 2022: the gene name in the preceding sentence has been corrected.]. (a) Relative expression of MdSnRK2s family genes during apple fruit development and ripening. (b) Screening for MdSnRK2 family members involved in regulating ETH production. MdSnRK2 genes were overexpressed in apple callus cells. ETH content was determined at 0, 12 and 24 h post‐infection with *Agrobacterium*. EV, empty vector. (c) Immunoblot analysis of MdSnRK2.4 and MdSnRK2.9 protein levels using specific antibodies to MdSnRK2.4 and MdSnRK2.9. Actin levels were used as an input control. (d) Phosphorylation level of MdSnRK2.4/2.9 throughout apple fruit development and ripening. Protein extracts were separated in a phos‐tag gel. Phosphorylated and nonphosphorylated MdSnRK2.4/2.9 were detected with anti‐MdSnRK2.4/2.9 antibody and quantitated by densitometry using ImageJ. In (a, b), values are means ± SD of three biological replicates. Different letters in (a) indicate significant differences according to Tukey’s honestly significant difference (HSD) test (*P* < 0.01). The asterisks in (b) indicate significant differences compared with the EV control (*, *P* < 0.05; **, *P* < 0.01; Student’s *t*‐test). DPA, d post‐anthesis. See also Supporting Information Figs S1 and S2.

We identified nine MdSnRK2 genes that belong to three subfamilies in apple calli (Fig. S1d). In particular, *MdSnRK2.1*, *MdSnRK2.4* and *MdSnRK2.9* had high expression levels, especially at 105 DPA (Fig. 1a). We overexpressed all *MdSnRK2s* into apple calli and measured ETH biosynthesis. Overexpression of the subfamily I members *MdSnRK2.4* and *MdSnRK2.9* (*MdSnRK2.4*‐OE and *MdSnRK2.9*‐OE) resulted in the highest ETH levels (Fig. 1b). This indicates that MdSnRK2.4 and MdSnRK2.9 are involved in the regulation of ETH biosynthesis in apple fruit cells. Notably, although expression levels of *MdSnRK2.4* and *MdSnRK2.9* were consistently high during the ripening process, they did not increase with the ripening process (Fig. 1a). We then detected the protein and phosphorylation levels of MdSnRK2.4 and MdSnRK2.9 during apple fruit ripening. Although MdSnRK2.4 and MdSnRK2.9 protein levels decreased slightly with ripening (Fig. 1c), their phosphorylation levels were significantly upregulated from 85 DPA and then gradually increased throughout the ripening process (Fig. 1d). These results were consistent with an increase in activated phosphorylation sites of MdSnRK2.4 and MdSnRK2.9 from 85 to 130 DPA (Fig. S2; Table S1). Taken together, our results indicate that MdSnRK2‐I regulates ETH biosynthesis via phosphorylation during fruit ripening.

### MdSnRK2.4 and MdSnRK2.9 regulate the expression of *MdACO1* and degradation of MdACS1

**Fig. 2:**
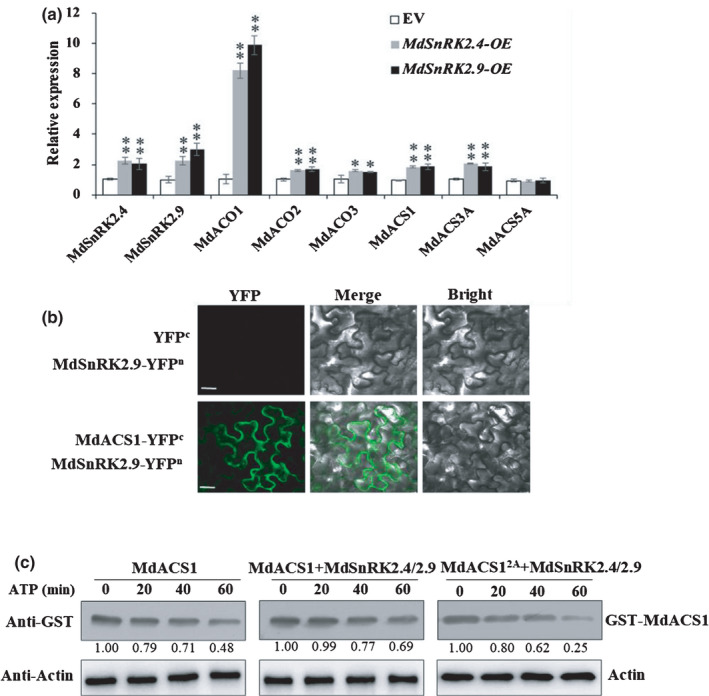
*Sucrose non‐ferment 1 related protein kinase 2* MdSnRK2.4 and MdSnRK2.9 regulate *MdACO1* expression and MdACS1 in apple (ACO, 1‐aminocyclopropane‐1‐carboxylic acid oxidase; ACS, 1‐aminocyclopropane‐1‐carboxylic acid synthase) [Correction after first publication 22 March 2022: the gene name in the preceding sentence has been corrected.]. (a) Expression of *MdACOs* and *MdACSs* in apple calli with overexpression (OE) of *MdSnRK2.4* and *MdSnRK2.9*. Data were normalized to *MdACTIN* transcript levels. Bars are means ± SD of three biological replicates. Asterisks denote significant differences from the empty vector (EV) control (*, *P* < 0.05; **, *P* < 0.01; Student’s *t*‐test). (b) Interactions between MdSnRK2.9 and MdACS1 determined by bimolecular fluorescence complementation (BiFC) analysis. Tobacco leaves were transiently transformed with MdSnRK2.9‐YFPn and MdACS1‐YFPc (YFP, yellow fluorescent protein). The physical interaction was examined by confocal laser‐scanning microscopy. Bars, 20 µm. (c) *In vitro* cell‐free degradation assay showing degradation of glutathione S‐transferase (GST)‐MdSnRK2.4/2.9 suppresses MdACS1 degradation. Equal amounts of purified MdSnRK2.4/2.9‐His were incubated with recombinant GST‐MdACS1 or GST‐MdACS^2A^ (MdACS^S5A S454A^) proteins in the presence of ATP. MdACS1 was detected with anti‐GST antibody and actin was used as a control.

1‐aminocyclopropane‐1‐carboxylic acid synthases (ACSs) and 1‐aminocyclopropane‐1‐carboxylic acid oxidases (ACOs) are the rate‐limiting enzymes in ethylene biosynthesis (Yang & Hoffman, 1984; Kende, 1993). To determine which genes are involved in MdSnRK2.4‐ and MdSnRK2.9‐meditated ETH biosynthesis, we analyzed the expression of *MdACOs* and *MdACSs* in *MdSnRK2.4*‐OE and *MdSnRK2.9*‐OE apple calli. The expression levels of *MdACO1*, *MdACO2*, *MdACO3*, *MdACS1* and *MdACS3A* were significantly higher in *MdSnRK2.4*‐OE and *MdSnRK2.9*‐OE calli than in control calli. Moreover, the expression levels of *MdACO1* were four‐ to six‐fold higher than those of other biosynthesis genes in *MdSnRK2.4*‐OE, *MdSnRK2.9*‐OE apple calli (Fig. 2a). *MdACO1* is the major *ACO* gene controlling ethylene biosynthesis in apple fruit (Nakatsuka *et al*., 1998; Schaffer *et al*., 2007). These results indicate that *MdACO1* is an important downstream biosynthesis gene affected by MdSnRK2.4 and MdSnRK2.9.

ACSs are involved in ETH biosynthesis via regulating their gene expression and phosphorylation‐mediated protein degradation (Park *et al*., 2021). We found that MdSnRK2.9 interacted with MdACS1 (Fig. 2b). The cell‐free degradation assay showed that co‐incubation of MdSnRK2.4‐His and MdSnRK2.9‐His could reduce the degradation of GST‐MdACS1. However, it did not obviously affect the degradation of GST‐MdACS1^2A^, the phospho‐dead mutant of MdACS1 that was generated by inactivating SnRK2‐recognized phosphorylation sites (Fig. 2c).

Compared to ACSs, the regulatory mechanisms of ACOs, in particular their post‐transcriptional regulatory mechanism, remain largely unknown. Therefore, we focused on determining how MdSnRK2‐I induces the expression of *MdACO1* to promote ETH biosynthesis.

### MdSnRK2.4 and MdSnRK2.9 are activated by osmotic stress and ETH in apple fruits

**Fig. 3:**
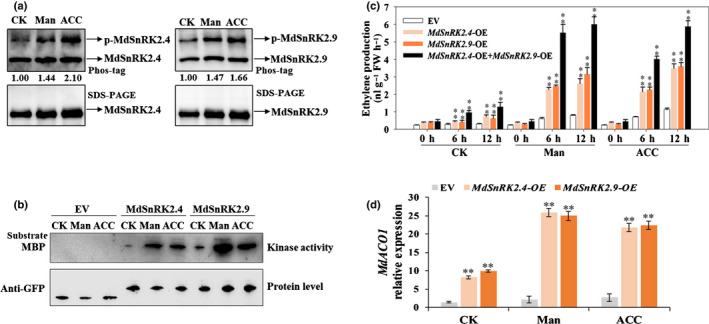
*Sucrose non‐ferment 1 related protein kinase 2* MdSnRK2.4 and MdSnRK2.9 are activated by ethylene (ETH) and mannitol in apple fruit [Correction after first publication 22 March 2022: the gene name in the preceding sentence has been corrected.]. (a) Phosphorylation level of MdSnRK2.4/2.9 in apple fruit flesh after mannitol and acetyl‐CoA carboxylase (ACC) treatment. Protein extracts were separated in a phos‐tag gel. Phosphorylated and nonphosphorylated MdSnRK2.4/2.9 were detected with anti‐MdSnRK2.4 or anti‐MdSnRK2.9 specific antibody and quantitated by densitometry using ImageJ. (b) Kinase activities of MdSnRK2.4 and MdSnRK2.9 in apple callus cells. Cells were transformed with vectors harboring green fluorescent protein (GFP)‐tagged *MdSnRK2.4* and *MdSnRK2.9*. After the genes were transiently expressed for 12 h, the cells were treated with 100 µM ACC for 6 h or 6% mannitol for 6 h and kinase activity was detected using an in‐gel kinase assay with MBP as the substrate. (c) Effects of *MdSnRK2.4* and *MdSnRK2.9* overexpression (OE) combined with osmotic stress (mannitol treatment) and ETH (ACC) treatment on ETH production in apple callus cells. Cells were transformed with overexpression vectors harboring *MdSnRK2.4*, *MdSnRK2.9* or both *MdSnRK2.4* and *MdSnRK2.9*. After the genes were transiently expressed for 12 h, the cells were treated with 100 µM ACC or 6% mannitol for 0, 6 or 12 h. Values are means ± SD of three biological replicates. Asterisks indicate significant difference compared with the empty vector (EV) control (**, *P* < 0.01; Student’s *t*‐test). (d) Effects of *MdSnRK2.4* and *MdSnRK2.9* overexpression in relation to osmotic stress (mannitol treatment) and ETH (ACC) treatment on *MdACO1* expression in apple callus cells. Values are means of three replicates ± SD. Asterisks indicate significant difference compared with the EV control (**, *P* < 0.01; Student’s *t*‐test). CK, no ACC or mannitol treatment; Man, mannitol treatment; ACC, ethylene precursor ACC treatment. See also Supporting Information Fig. S3.

As MdSnRK2.4 and MdSnRK2.9 are activated during fruit ripening (Fig. 1d), we next sought to determine whether these proteins are activated by ETH. We investigated the effects of ACC treatment on phosphorylation changes in MdSnRK2.4 and MdSnRK2.9 in apple flesh (Fig. 3a).

The SnRK2 family is reported to be widely involved in osmotic stress, which, in turn, induces ETH biosynthesis (Skirycz *et al*., 2011; Zhang *et al*., 2011; Fujii & Zhu, 2012; Dubois *et al*., 2018). Therefore, we investigated the effects of mannitol treatment on MdSnRK2.4 and MdSnRK2.9. A phos‐tag assay showed that the phosphorylation levels of MdSnRK2.4 and MdSnRK2.9 in apple flesh were higher under 6% mannitol and 100 µM ACC treatments than under the control treatment at 105 DPA (Figs 3a, S3a–c). Additionally, an in‐gel assay revealed that MdSnRK2.4 and MdSnRK2.9 were highly activated in *MdSnRK2.4*‐OE and *MdSnRK2.9*‐OE apple calli under 6% mannitol and 100 µM ACC treatments (Fig. 3b).

Then, we determined whether the mannitol‐ and ACC‐activated MdSnRK2.4 and MdSnRK2.9 could mediate ETH biosynthesis in apple calli. As shown in Fig. 3(c), ETH production in *MdSnRK2.4*‐OE and *MdSnRK2*.*9*‐OE apple calli was significantly higher under 6% mannitol and 100 µM ACC treatments compared with that under the control treatment. Moreover, co‐transformed *MdSnRK2.4*‐OE and *MdSnRK2.9*‐OE apple calli had higher ETH levels than single transformation calli (Fig. 3c). These results indicate that mannitol‐ and ACC‐activated MdSnRK2.4 and MdSnRK2.9 synergistically regulate ETH biosynthesis in apple fruit.

Notably, *MdACO1* expression in *MdSnRK2.4*‐OE and *MdSnRK2.9*‐OE apple calli was more sensitive to 6% mannitol and 100 µM ACC than that in EV fruit calli, indicating that *MdACO1* expression is the important output for MdSnRK2.4‐ and MdSnRK2.9‐mediated ETH biosynthesis under mannitol and ACC treatments (Fig. 3d).

### MdSnRK2.4 and MdSnRK2.9 interact with and phosphorylate MdHB1 and MdHB2

**Fig. 4:**
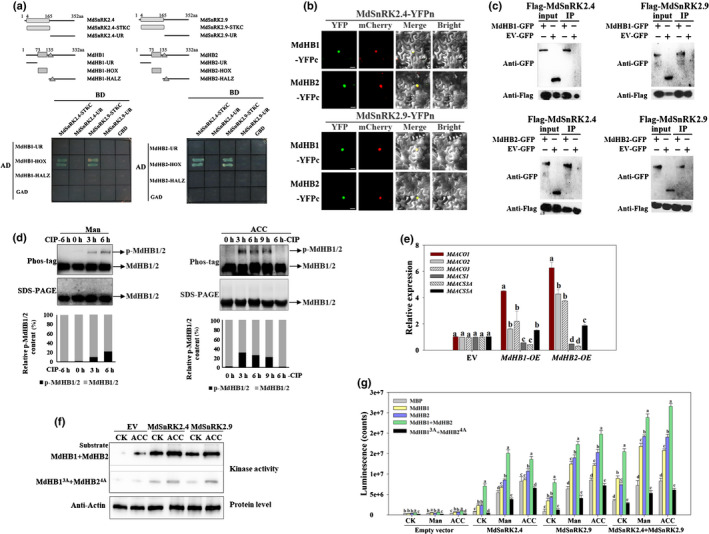
*Sucrose non‐ferment 1 related protein kinase 2* MdSnRK2.4 and MdSnRK2.9 phosphorylate MdHB1 and MdHB2 *in vitro* and *in vivo* [Correction after first publication 22 March 2022: the gene name in the preceding sentence has been corrected.]. (a) Upper panel, diagrams showing the MdSnRK2.4, MdSnRK2.9, MdHB1 and MdHB2 regions that were assessed. Lower panel, yeast‐two‐hybrid analysis of the interactions between the different protein regions. (b) Bimolecular fluorescence complementation (BiFC) analysis of the physical interactions of MdSnRK2.4 and MdSnRK2.9 with MdHB1 and MdHB2 in tobacco leaves transiently transformed with *MdSnRK2.4‐YFPn* or *MdSnRK2.9‐YFPn* and *MdHB1YFPc* or *MdHB2‐YFPc* (YFP, yellow fluorescent protein). Physical interactions were examined by confocal laser‐scanning microscopy. Bars, 20 µm. (c) Co‐immunoprecipitation (Co‐IP) analysis of the physical interactions of MdSnRK2.4 and MdSnRK2.9 with MdHB1 and MdHB2 in protein extracts of apple callus cells harboring FLAG‐tagged *MdSnRK2.4* or *MdSnRK2.9* and green fluorescent protein (GFP)‐tagged *MdHB1* or *MdHB2*. FLAG‐tagged MdSnRK2.4 and MdSnRK2.9 were immunoprecipitated with anti‐FLAG immunoglobulin (Ig)G and the co‐immunoprecipitated proteins were blotted with anti‐GFP IgG. An empty vector (EV) pMDC83 carrying GFP was used as a control. (d) Phosphorylation level of MdHB1/2 in apple fruit flesh after acetyl‐CoA carboxylase (ACC) or mannitol treatment for the indicated periods. Protein extracts were separated in a phos‐tag gel. Phosphorylated and nonphosphorylated MdHB1/2 were detected with anti‐MdHB1/2 antibody and quantitated by densitometry using ImageJ. Percentages were calculated. Phosphorylated MdHB1/2 proteins clearly decreased after a calf‐intestinal alkaline phosphatase (CIP) treatment. (e) Expression of *MdACOs* and *MdACSs* as affected by the overexpression (OE) of *MdHB1* and *MdHB2* (ACO, 1‐aminocyclopropane‐1‐carboxylic acid oxidase; ACS, 1‐aminocyclopropane‐1‐carboxylic acid synthase). *MdHB1* and *MdHB2* were transiently expressed in apple callus cells. Data were normalized to the *MdACTIN* transcript level. Bars are means ± SD of three biological replicates. Different letters indicate significant difference by Tukey’s honestly significant difference (HSD) test (*P* < 0.01). (f) In‐gel assays of the kinase activity of MdSnRK2.4 and MdSnRK2.9 on their MdHB1 and MdHB2 substrates in response to ethylene stimulation. Apple callus transiently expressing GFP*‐*tagged *MdSnRK2.4* or *MdSnRK2.9* was treated with 100 µM ACC for 6 h, and kinase activity was detected by in‐gel kinase activity assays with purified MdHB1/2 and mutated MdHB1/2 as substrates. The superscripts ‘MdHB1^3A^’and ‘MdHB2^4A^’ denote Ala substitution of the Thr‐81, Thr‐94 and Thr‐130 phosphorylation sites for MdHB1; Thr83, Ser84, Thr96 and Thr132 phosphorylation sites for MdHB2, respectively. Empty vector, apple callus transformed with empty vector. CK, nontreatment control. (g) Kinase activity of MdSnRK2.4 and MdSnRK2.9 on their MdHB1 and MdHB2 substrates in response to mannitol and ACC treatment. Apple callus cells transiently expressing *GFP‐*tagged *MdSnRK2.4* or *MdSnRK2.9* were treated with 100 µM ACC or 6% mannitol for 6 h. Kinase activity was detected using the ADP‐Glo™ kinase assay protocol with MBP, purified MdHB1‐His, MdHB2‐His, combined mutated or nonmutated MdHB1‐His, and MdHB2‐His as substrates. Bars are means ± SD of three biological replicates. Different letters indicate significant difference by Tukey’s HSD test (*P* < 0.01). CK, no ACC or mannitol treatment; Man, mannitol treatment; ACC, ethylene precursor ACC treatment. See also Supporting Information Fig. S4 and Table S2.

In order to determine how MdSnRK2.4 and MdSnRK2.9 regulate *MdACO1* expression, we investigated the substrates of MdSnRK2‐I by IP‐MS. More than 30 candidate proteins were identified, including one transcription factor (ID: V5LLY3) (Table S2). This transcription factor belongs to subfamily I of the HD‐Zip family and has a high sequence similarity with LeHB‐1, the reported regulator of *ACO1* in tomato fruits (Fig. S4a,b; Lin *et al*., 2008). We named this transcription factor MdHB1. Then, we performed Y2H to examine how MdHB1 interacts with MdSnRK2.4 and MdSnRK2.9 (Fig. 4a). MdSnRK2.4 and MdSnRK2.9 interacted with the HOX (Homeobox) domain of MdHB1 via the kinase activity domain (Fig. 4a). Therefore, we identified 23 HD‐Zip I members in apple and determined their expression patterns in apple fruit during ripening. Of the HD‐Zip I members detected, MdHB1 and MdHB2 had the highest expression levels (Fig. S4c,d). Additionally, more ETH was produced in *MdHB1‐*OE and *MdHB2*‐OE apple calli than in EV apple calli (Fig. S4e). A BiFC assay showed that MdSnRK2.4 and MdSnRK2.9 interacted only with MdHB1 and MdHB2, and not with the other five HD‐Zip I proteins detected (Figs 4b, S4e,f). We also validated these interactions using Co‐IP by expressing *MdHB1/2‐GFP* and *FLAG‐MdSnRK2.4/2.9* in apple calli (Fig. 4c). Taken together, these results indicate that MdSnRK2.4 and MdSnRK2.9 interact with MdHB1 and MdHB2 at their kinase activity domain in the nuclei of apple fruit (Fig. 4a–c, S4a–g).

The phos‐tag assay showed that the phosphorylation levels of MdHB1 and MdHB2 in apple calli also were increased by 6% mannitol and 100 µM ACC treatments (Fig. 4d). The expression level of *MdACO1* in *MdHB1*‐OE and *MdHB2*‐OE apple calli was higher than those of other ETH biosynthesis genes, which is consistent with the *MdACO1* expression pattern in *MdSnRK2.4*‐OE and *MdSnRK2.9*‐OE apple calli (Figs 2a, 4e). These results suggest that MdHB1 and MdHB2 are involved in mannitol‐ and ACC‐induced ethylene biosynthesis as the substrates of MdSnRK2.4 and MdSnRK2.9 (Figs 4d, S4b,h,i). We then examined whether MdSnRK2.4 and MdSnRK2.9 can phosphorylate MdHB1 and MdHB2 *in vitro* (Fig. 4f,g). MdSnRK2.4‐eGFP and MdSnRK2.4‐eGFP immunocomplexes, which were purified from *MdSnRK2.4*‐OE and *MdSnRK2.9*‐OE single‐ or co‐transformed apple calli, phosphorylated MdHB1‐His, MdHB2‐His and their mixed proteins *in vitro* (Fig. 4f,g). The 6% mannitol and 100 µM ACC treatments enhanced MdSnRK2.4‐ and MdSnRK2.9‐mediated phosphorylation levels of MdHB1‐His, MdHB2‐His and their mixed proteins (Fig. 4g). These results indicate that MdHB1 and MdHB2 act as substrates in a synergistic response to mannitol‐ and ACC‐activated MdSnRK2.4 and MdSnRK2.9.

We further examined the phosphorylation sites of MdMdHB1‐His and MdHB2‐His induced by GST‐MdSnRK2.9 using MS technology and found that MdSnRK2.9 phosphorylated MdHB1 at sites T81, T94 and T130, and MdHB2 at sites T83, S84, T96 and T132 (Fig. S4j,k). Then, we mutated all of the threonine and serine in these sites to alanine to generate the phospho‐dead proteins MdHB1^T81A,T94A,T130A^ (MdHB1^3A^) and MdHB2^T83A,S84A,T96A,T132A^ (MdHB2^4A^). Under the control treatment, the phosphorylation levels of MdHB1^3A^‐His and MdHB2^4A^‐His were increased slightly by MdSnRK2.4‐eGFP and MdSnRK2.9‐eGFP either alone or in combination (Fig. 4f,g). The 6% mannitol and 100 µM ACC treatments also increased the phosphorylation levels of MdHB1^3A^‐His and MdHB2^4A^‐His, but to a lesser extent than those observed for MdHB1‐His and MdHB2‐His (Fig. 4f,g). These results indicate that T81, T94 and T130 were the corresponding phosphorylation sites of MdHB1, and that T83, S84, T96 and T132 were those of MdHB2 for mannitol‐ and ACC‐activated MdSnRK2‐I. However, MdHB1^3A^‐His and MdHB2^4A^‐His were not completely inactivated, suggesting that MdHB1 and MdHB2 have other phosphorylation sites involved in MdSnRK2‐I‐mediated osmotic stress and ETH responses (Fig. 4f,g).

### MdHB1 and MdHB2 are the downstream substrates of MdSnRK2.4 and MdSnRK2.9 and regulate *MdACO1* expression to mediate ETH biosynthesis

**Fig. 5:**
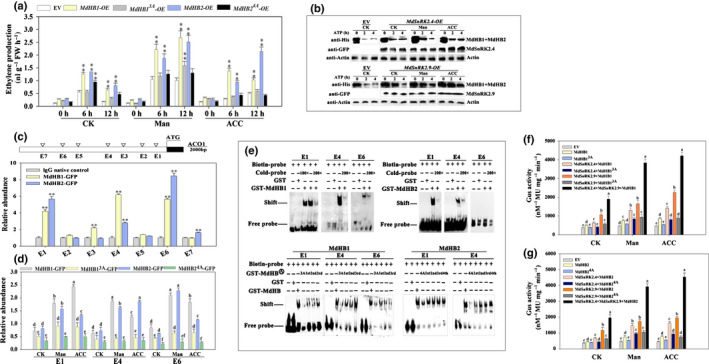
MdHB1 and MdHB2 act as the downstream substrates of *sucrose non‐ferment 1 related protein kinase 2* MdSnRK2.4 and MdSnRK2.9 in apple by regulating *MdACO1* expression to mediate ethylene (ETH) production (ACO, 1‐aminocyclopropane‐1‐carboxylic acid oxidase) [Correction after first publication 22 March 2022: the gene name in the preceding sentence has been corrected.]. (a) Effect of *MdHB1* and *MdHB2* overexpression (OE) on ETH production in apple callus cells. Cells were transformed with overexpression vectors harboring *MdHB1*, *MdHB2*, mutated *MdHB1* or mutated *MdHB2*. After the genes were transiently expressed for 12 h, the cells were treated with 100 µM acetyl‐CoA carboxylase (ACC) or 6% mannitol for 0, 6, or 12 h. (b) MdSnRK2.4/2.9 suppresses MdHB1/2 degradation. *In vitro* cell‐free degradation assays were performed. *MdSnRK2.4* and *MdSnRK2.9* were transiently expressed in apple callus cells, which then were treated with ACC or mannitol. Equal amounts of total proteins were extracted and incubated with recombinant MdHB1/2‐His proteins in the presence of ATP. MdHB1/2 were detected with anti‐His antibody and actin was used as a control. (c) Chromatin immunoprecipitation (ChIP)‐PCR analysis for identification of the MdHB1‐ and MdHB2‐binding elements in the *MdACO1* promoter. Green fluorescent protein (GFP)*‐*fused *MdHB* genes (*MdHB1‐GFP* and *MdHB2‐GFP*) were transiently expressed in apple callus cells, and the MdHB1 and MdHB2 proteins were immunoprecipitated with anti‐GFP immunoglobulin (Ig)G for ChIP‐PCR analysis of the predicted binding elements in the *MdACO1* promoter. Nonimmune IgG was used as the negative control. E1, recognition element of transcription; E2, cis‐acting regulatory element involved in auxin responsiveness; E3, cis‐acting element involved in the abscisic acid responsiveness; E4, TC‐rich defense‐ and stress‐responsive element. E5, MYB binding site; E6, ethylene‐responsive element; E7, salicylic acid responsive element. (d) ChIP‐PCR analysis of the mutated MdHB1 and MdHB2 binding activity to E1, E4 and E6 elements in the *MdACO1* promoter. Apple callus cells expressing GFP‐fused *MdHB* genes (*MdHB1‐GFP*, *MdHB1^3A^‐GFP, MdHB2‐GFP* and *MdHB2^4A^‐GFP*) were treated with 6% mannitol or 100 µM ACC for 6 h. Chromatin was purified from the transgenic calli and immunoprecipitated with anti‐GFP antibody or without antibody. The amounts of indicated DNA in the immune complex were determined by quantitative reverse transcription (qRT)‐PCR. An *actin* fragment was amplified as a control. Relative enrichment was calculated as input % of the indicated DNA/input % of control. (e) Electrophoretic mobility‐shift assay (EMSA) for further confirmation of the binding activity of MdHB1 and MdHB2 to *MdACO1* promoter elements. Different constitutively active mutants of MdHB1 and MdHB2 were constructed by replacing the threonine and serine residues at the phosphorylation sites (Thr^81^, Thr^94^, and Thr^130^ for MdHB1; Thr^83^, Ser^84^, Thr^96^, and Thr^132^ for MdHB2) with alanine residues, which mimic a nonphosphorylatable mutation. ‘3A’ or ‘4A’ denotes total mutation of all phosphorylation sites. ‘1st’, ‘2nd’, ‘3rd’ and ‘4th’ denote the mutation of the first, second, third and fourth phosphorylation sites, respectively. (f, g) MdSnRK2.4‐ and MdSnRK2.9‐mediated signaling cascade. Different combinations of the signaling effectors were incorporated into the pCambia1301 vector, which contained the *GUS* gene driven by the *MdACO1* promoter as a reporter. The vectors harboring the indicated combination of effector (*MdSnRK2.4*, *MdSnRK2.9*, *MdHB1* and *MdHB2*) and reporter genes were introduced into *Agrobacterium tumefaciens*, which then was transformed into apple callus cells for a stimulation–response analysis. Stimulation was performed through ACC treatment or osmotic stress, after which the activity of the GUS reporter was examined. In (a, c, d, f, g), values are means ± SD of three biological replicates. Different letters in (d, f, g) indicate significant differences according to Tukey’s honestly significant difference (HSD) test (*P* < 0.01). The asterisks in (a, c) indicate significant differences compared with the control (**, *P* < 0.01; Student’s *t*‐test). CK, no ACC or mannitol treatment; Man, mannitol treatment; ACC, ETH precursor ACC treatment.

In order to determine the effects of phosphorylation on the biological functions of MdHB1 and MdHB2, we overexpressed *MdHB1*‐eGFP, *MdHB2*‐eGFP, *MdHB1^3A^*‐eGFP and *MdHB2^4A^*‐eGFP in apple calli and measured ETH biosynthesis. *MdHB1*‐OE and *MdHB2*‐OE apple calli had significantly higher ETH biosynthesis levels than EV calli, particularly under 6% mannitol and 100 µM ACC treatments. However, ETH production in *MdHB1^3A^*‐OE and *MdHB2^4A^*‐OE apple calli was not significantly different from that in EV calli, except for that in *MdHB2^4A^*‐OE calli under the control treatment and *MdHB3^4A^*‐OE calli under the mannitol treatment (Fig. 5a).

In general, phosphorylation can regulate the biological function of transcription factors by altering their stability or transcriptional activity (Liu *et al*., 2017; Srivastava *et al*., 2020). Therefore, we determine whether phosphorylation could change the stability of MdHB1 and MdHB2 proteins. Incubation with protein extractions from *MdSnRK2.4*‐OE and *MdSnRK2.9*‐OE apple calli reduced the degradation rate of MdHB1 and MdHB2 mixed proteins (Fig. 5b). This was enhanced under mannitol and ACC treatments. Then, we determined the effects of phosphorylation on the transcriptional activity of MdHB1 and MdHB2 using ChIP‐PCR. MdHB1 and MdHB2 interacted with the promoter of *MdACO1* by binding several elements. Among these elements, E1 (recognition element of transcription), E4 (TC‐rich defense and stress‐responsive element) and E6 (ETH‐responsive element) were used to further explore the interaction with MdHB1^3A^ and MdHB2^4A^ (Fig. 5c). ACC and mannitol treatments enhanced the interactions of MdHB1 and MdHB2, but not those of MdHB1^3A^ and MdHB2^4A^ with E1, E4 and E6 (Fig. 5d). Moreover, an EMSA showed that MdHB1 and MdHB2 directly bound E1, E4 and E6 *in vitro* and that the binding with MdHB1^3A^ and MdHB2^4A^ was weaker (Fig. 5e). These results indicate that MdSnRK2.4‐ and MdSnRK2.9‐mediated phosphorylation is important for the protein stability and transcriptional activity of MdHB1 and MdHB2 (Fig. 5b–e).

Next, we investigated the signaling transduction pathway of MdSnRK2.4‐ and MdSnRK2.9‐mediated ETH biosynthesis. We overexpressed *MdSnRK2.4*, *MdSnRK2.9*, *MdHB1*, *MdHB2, MdHB1^3A^* and *MdHB2^4A^* to determine their effects on *MdACO1* expression in apple calli. *MdACO1* expression was significantly higher in *MdSnRK2.4*‐OE, *MdSnRK2.9*‐OE, *MdHB1*‐OE, *MdHB2*‐OE and their kinase‐substrate co‐transformed combinations than in EV calli (Fig. 5f,g). However, under mannitol and ACC treatments, the *MdACO1* expression in *MdHB1^3A^ MdHB2^4A^* calli, either with or without *MdSnRK2.4*‐OE and *MdSnRK2.9*‐OE, was either similar, or only slightly higher, than that in EV calli (Fig. 5f,g). These results indicate that osmotic stress‐ and ETH‐activated MdSnRK2.4 and MdSnRK2.9 can phosphorylate MdHB1 at sites T81, T94 and T130, and MdHB2 at sites T83, S84, T96 and T132. This phosphorylation enhanced their protein stability and transcriptional activity to regulate *MdACO1* expression and therefore control ETH biosynthesis in apple fruit (Figs 1, 2, 3, 4, 5).

### MdSnRK2‐I and SlSnRK2‐I regulate tomato fruit ripening by mediating ETH biosynthesis

**Fig. 6:**
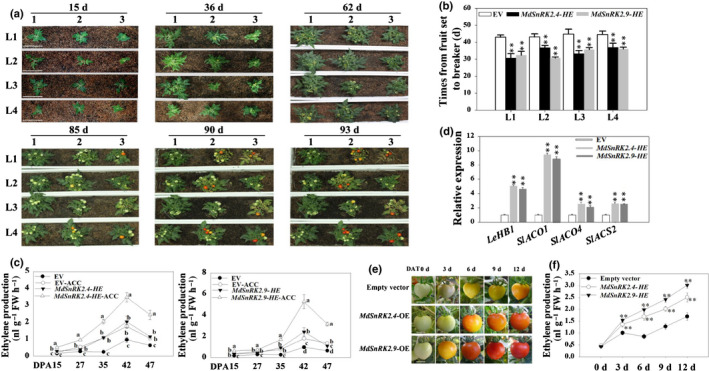
*Sucrose non‐ferment 1 related protein kinase 2*‐related protein kinases MdSnRK2.4 and MdSnRK2.9 regulate tomato fruit ripening by mediating ethylene (ETH) biosynthesis [Correction after first publication 22 March 2022: the gene name in the preceding sentence has been corrected.]. (a) Effect of *MdSnRK2.4* and *MdSnRK2.9* heterologous expression (HE) in tomato plants. Tomato plants were stably transformed with *MdSnRK2.4* and *MdSnRK2.9*. Seeds of the T_3_ generation were geminated and transplanted into soil. Plant growth and development were observed until fruit ripening. For each transgenic plant, four independent lines are shown; days after seed germination is marked above each photograph. 1, empty vector (EV); 2, *MdSnRK2.4‐HE*; 3, *MdSnRK2.9‐HE*. Bars, 10 cm. (b) Time from fruit set to breaker in the different *MdSnRK2.4* and *MdSnRK2.9* transgenic lines. *n* = 12. Values are means ± SD and asterisks denote significant differences between transgenic and EV plants (**, *P* < 0.01; Student’s *t*‐test). (c) Effect of heterologous *MdSnRK2.4* and *MdSnRK2.9* in relation to ethylene (acetyl‐CoA carboxylase, ACC) treatment on ETH production in transgenic tomato fruit. Values are means ± SD of three biological replicates. Different letters denote significant differences (*P* < 0.01) among samples as determined by Tukey’s honestly significant (HSD) test. ACC, denotes the tomatoes were treated with ACC. (d) Effect of *MdSnRK2.4* and *MdSnRK2.9* on the expression of ETH‐ and ripening‐related genes. Quantitative reverse transcription (qRT)‐PCR was conducted using *SlACTIN* as an internal control. Values are means ± SD of three biological replicates. Asterisks denote significant differences between transgenic and EV plants (**, *P* < 0.01; Student’s *t*‐test). (e, f) Effect of transient *MdSnRK2.4* and *MdSnRK2.9* heterologous expression (HE) on fruit ripening (e) and ETH production (f). The two genes were cloned individually into the pCambia1304 expression vector, in which the target gene was driven by the *35S* promoter. Transient gene expression was conducted by injecting the fruit with *Agrobacterium tumefaciens* harboring the expression vectors or an empty vector (EV; control). Ethylene content was measured 0, 3, 6, 9 and 12 d post‐infection. The experiments were repeated three times with similar results. Values are means ± SD of three biological replicates. Asterisks indicate significant differences between transgenic and EV plants (**, *P* < 0.01; Student’s *t*‐test). DAT, days after transfection. DPA, days post‐anthesis. Bars, 1 cm. See also Supporting Information Fig. S5.

In order to further explore the roles of MdSnRK2‐I in climacteric fruit ripening, four independent *MdSnRK2.4*‐*HE* (*heterologous expression*) and *MdSnRK2.9*‐*HE* transgenic tomato lines were generated (Fig. S5a). Compared to EV fruit, ripening was significantly accelerated and ethylene biosynthesis was significantly increased in *MdSnRK2.4*‐*HE* and *MdSnRK2.9*‐*HE* fruit (Figs 6a,b, S5b). Similar to the effect in apple fruit, a 100 µM ACC treatment increased ETH biosynthesis in *MdSnRK2.4*‐*HE* and *MdSnRK2.9*‐*HE* tomato fruit to a greater extent than in EV fruit (Fig. 6c).

We then determined the expression levels of *HB*/*ACO* module genes in *MdSnRK2.4*‐*HE* and *MdSnRK2.9*‐*HE* tomato fruit. *SlACO1*, *SlACO4* and *SlHB1*(*LeHB‐1*) expression levels were significantly higher in *MdSnRK2.4*‐*HE* and *MdSnRK2.9*‐*HE* tomato fruit than in EV fruit (Fig. 6d). These three genes are important biosynthesis genes controlling ETH‐mediated tomato fruit ripening (Nakatsuka *et al*., 1998; Cara & Giovannoni, 2008; J. Liu *et al*., 2015; M. Liu *et al*., 2015). In addition, compared with EV plants, *MdSnRK2.4*‐*HE* and *MdSnRK2.9*‐*HE* tomato plants were dwarfed, had twisted leaves and had shorter internodes with more branches (Fig. S5c,d). This is a typical phenotype associated with high levels of ETH (Li *et al*., 2021; Pattyn *et al*., 2021). Additionally, the leaves of transgenic plants turned yellow earlier than those of EV plants, indicating that *MdSnRK2.4* and *MdSnRK2.9* heterologous expression accelerated tomato plant senescence (Fig. 6a). Because stable transgenic expression affected plant growth, we transiently expressed *MdSnRK2.4* and *MdSnRK2.9* in tomato fruit to isolate the effect of MdSnRK2‐I on fruit ripening. The ETH content was significantly higher in *MdSnRK2.4*‐*HE* and *MdSnRK2.9*‐*HE* fruit than in EV fruit, and ripening was faster (Fig. 6e,f). These results suggest that *MdSnRK2.4*‐*HE* and *MdSnRK2.9*‐*HE* regulate tomato fruit ripening by affecting HB/ACO module‐mediated ETH biosynthesis.

**Fig. 7:**
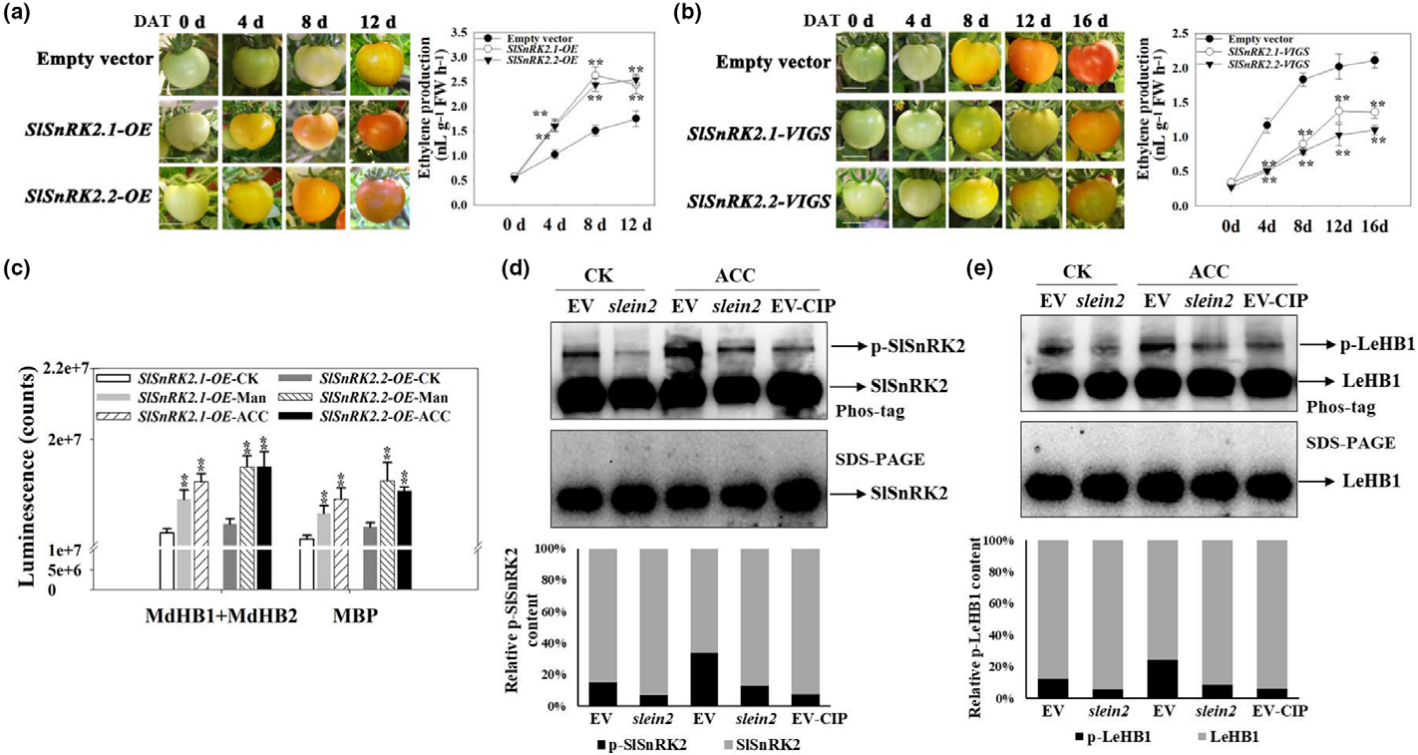
*Sucrose non‐ferment 1 related protein kinase 2* SlSnRK2‐I regulates ethylene (ETH)‐mediated tomato fruit ripening [Correction after first publication 22 March 2022: the gene name in the preceding sentence has been corrected.]. (a) Effect of transient *SlSnRK2.1* and *SlSnRK2.2* overexpression (OE) on tomato fruit ripening and ETH production. The two genes were cloned individually into the pCambia1304 expression vector, in which the target gene was driven by the 35S promoter. Transient gene expression was conducted by injecting the fruit with *Agrobacterium tumefaciens* strains harboring expression vectors, or with a strain harboring the empty vector (EV) as a control. The experiments were repeated three times with similar results. DAT, days after transfection. ETH content was measured at the indicated time points after infection. [Correction after first publication 22 March 2022: panel (a) in the figure has been updated.] (b) Effect of *SlSnRK2.1/2.2*‐VIGS (virus‐induced gene silencing) on tomato fruit ripening and ETH production. (c) Kinase activity of SlSnRK2.1 and SlSnRK2.2 on their MBP, MdHB1 and MdHB2 substrates in response to mannitol and acetyl‐CoA carboxylase (ACC) treatment. Tomato fruits transiently overexpressing *SlSnRK2.1* or *SlSnRK2.2* were treated with 100 µM ACC or 6% mannitol for 6 h, and kinase activity was detected using the ADP‐Glo™ kinase assay protocol with MBP, MdHB1 and MdHB2 as substrates. (d) Phosphorylation level of SlSnRK2 in *SlEIN2‐VIGS* tomato fruit after ACC treatment (EIN2, ethylene‐insensitive protein 2). The phosphorylation level of SlSnRK2 was detected by immunoblotting using antibodies to SlSnRK2. Protein extracts were separated in a phos‐tag gel, and the phosphorylated and nonphosphorylated SlSnRK2 proteins were detected. Images were quantitated by densitometry using ImageJ, and percentages were calculated. Phosphorylated SlSnRK2 proteins clearly decreased after a calf‐intestinal alkaline phosphatase (CIP) treatment. (e) Phosphorylation level of LeHB1 in *SlEIN2‐VIGS* tomato fruit after ACC treatment. The phosphorylation level of LeHB1 was detected by immunoblotting using antibodies to LeHB1. Protein extracts were separated in a phos‐tag gel, and the phosphorylated and nonphosphorylated LeHB1 proteins were detected. Images were quantitated by densitometry using ImageJ, and percentages were calculated. Phosphorylated proteins clearly decreased after a CIP treatment. In (a–c), asterisks indicate significant difference compared with the EV or CK control (**, *P* < 0.01; Student’s *t*‐test). DAT, days after transfection. Bars: (a, b) 1 cm. CK, no ACC or mannitol treatment; Man, mannitol treatment; ACC, ETH precursor ACC treatment. See also Supporting Information Fig. S5.

Based on our findings, we hypothesize that SnRK2‐I and the HB/ACO module may conservatively regulate ethylene biosynthesis in both apple and tomato fruits (Figs 1, 2, 3, 4, 5, 6). Eight SnRK2 members have been identified in tomato, of which SlSnRK2.1 and SlSnRK2.2 are members of subfamily I (Fig. S1d; Sun *et al*., 2011). To determine the function of *SlSnRK2.1* and *SlSnRK2.2*, we upregulated the expression of *SlSnRK2.1* and *SlSnRK2.2* using transient overexpression technology in tomato fruit. *SlSnRK2.1*‐OE and *SlSnRK2.2*‐OE fruit ripened faster and had significantly higher ETH production than EV fruit, whereas *SlSnRK2.1*‐VIGS and *SlSnRK2.2*‐VIGS fruit ripened slower and produced significantly less ETH than EV fruit (Fig. 7a,b). These results indicate that the SlSnRK2‐I subfamily is involved in regulating ETH biosynthesis and tomato fruit ripening. MdHB1, MdHB2 and their homolog *SlHB1* (*LeHB‐1*) all belong to the HD‐Zip I subfamily (Fig. S4a). We blasted two conserved peptides of MdHB1 and MdHB2 bound with MdSnRK2‐I, and three conserved phosphorylation sites of MdHB1 and MdHB2 mediated by MdSnRK2‐I against the tomato genome. These conserved peptides and phosphorylation sites were carried only by LeHB‐1, and not by other HD‐Zip I subfamily members (Fig. S4b). We also found that SlSnRK2.1 and SlSnRK2.2, which were activated by mannitol and ACC treatments, could phosphorylate MdHB1 and MdHB2 *in vitro* (Fig. 7c). This suggests that the phosphorylation regulatory mechanisms of SlSnRK2‐I and MdSnRK2‐I in response to mannitol and ACC treatments might be specific for particular substrate structures.

Ethylene‐insensitive protein 2 is an important component controlling endogenous ETH signaling and thus regulating fruit ripening (Fu *et al*., 2005; Gao *et al*., 2016). To determine whether endogenous ETH affects the function of the SnRK2‐HB module, we determined phosphorylation levels of the SnRK2 family and SlHB1 (LeHB‐1) in *SlEIN2*‐VIGS tomato fruit. Phosphorylation levels of SnRK2 and SlHB1 (LeHB‐1) were reduced in *SlEIN2*‐VIGS fruit compared with in EV fruit, both with and without ACC treatment (Figs 7d,e, S5d). These results suggested that the SlSnRK2‐SlHB1 module acts downstream of SlEIN2 in tomato fruit.

## Discussion

**Fig. 8:**
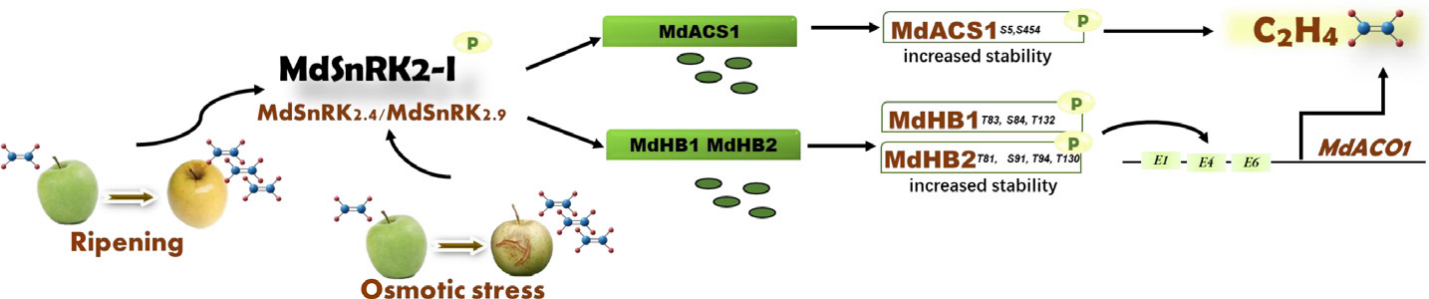
The *Sucrose non‐ferment 1 related protein kinase 2* (MdSnRK2)‐I/HB1 signaling module regulates *MdACO1*‐mediated ethylene (ETH) production (ACO, 1‐aminocyclopropane‐1‐carboxylic acid oxidase) [Correction after first publication 22 March 2022: the gene name in the preceding sentence has been corrected.]. MdSnRK2‐I members MdSnRK2.4 and MdSnRK2.9 were activated during fruit ripening or under osmotic stress and then phosphorylated the corresponding sites of MdHB1 and MdHB2 transcription factors to increase their stability and transcriptional activity. This enhanced the expression of their downstream gene *MdACO1*, encoding the rate‐limiting enzyme in ETH biosynthesis in apple fruit. MdSnRK2.4 and MdSnRK2.9 also interacted with another rate‐limiting enzyme, 1‐aminocyclopropane‐1‐carboxylic acid synthase (MdACS1), and enhanced its stability mediated by two phosphorylation‐sites. The increased *MdACO1* gene expression and enhanced MdACS1 protein stability increased ETH biosynthesis to promote ripening and respond to osmotic stress.

ETH is an important plant hormone with various biological functions. The regulatory mechanisms of ETH signal transduction and biosynthesis have been well‐established in *Arabidopsis thaliana* and crops such as maize (*Zea mays*) and rice (*Oryza sativa*) (Yang *et al*., 2015; Park *et al*., 2021). However, despite being the major hormone regulating the ripening of climacteric fruits, the regulation of its signal transduction and biosynthesis in fruit is largely unclear. In this study, we revealed a pathway mediated by phosphorylation that regulates ETH biosynthesis in apple fruit (Fig. 8). As shown in Fig. 8, the ABA‐unresponsive subfamily I of sucrose nonfermenting protein kinase 1‐related protein kinase 2 (MdSnRK2‐I) members MdSnRK2.4 and MdSnRK2.9 were activated during fruit ripening and under osmotic stress. Then, they phosphorylated specific sites of MdHB1 and MdHB2 transcription factors to increase their stability and transcriptional activity, thereby enhancing the expression of their downstream *1‐aminocyclopropane‐1‐carboxylic acid oxidase* gene (*MdACO1*), which encodes the rate‐limiting enzyme of ETH biosynthesis in apple fruit. In addition to *MdACO1*, MdSnRK2.4 and MdSnRK2.9 interacted with another rate‐limiting enzyme, 1‐aminocyclopropane‐1‐carboxylic acid synthase 1 (MdACS1), enhancing its stability by phosphorylating two sites. The higher *MdACO1* expression and enhanced MdACS1 protein stability increased ETH production, promoting ripening and mediating the plant's response to osmotic stress (Fig. 8).

SnRK2‐I, SnRK2‐II and SnRK2‐III modulate plant growth and abiotic stress responses, in particular, responses to drought‐ and salt‐triggered osmotic stress (Zhang *et al*., 2011; Fujii & Zhu, 2012; Fujita *et al*., 2013; Lin *et al*., 2020). Compared to SnRK2‐II and SnRK2‐III, little is known about the function, substrates and regulatory mechanisms of SnRK2‐I in higher plants (Maszkowska *et al*., 2021). SnRK2‐I is involved in responses to salt and osmotic stress by phosphorylating VARICOSE (VCS) and EARLY RESPONSE TO DEHYDRATION (ERD); however, its response to ABA was unknown (Fujii *et al*., 2011; Krzywińska *et al*., 2016; Lin *et al*., 2020). Additionally, it was unclear whether and how SnRK2‐I, SnRK2‐II, and SnRK2‐III are related to other plant hormones that function in osmoregulation (Maszkowska *et al*., 2021). Osmotic stress promotes ETH biosynthesis, and *ACOS* are important genes in this response (Skirycz *et al*., 2011; Zhang *et al*., 2016; Dubois *et al*., 2017, 2018; Savada *et al*., 2017). Our results indicate that osmotic stress activates MdSnRK2‐I to increase *MdACO1*‐mediated ETH biosynthesis in apple fruit (Fig. 3). These findings extend our understanding of the interactions between SnRK2s and plant hormones, as well as osmoregulation in higher plants. We also found that SlSnRK2‐I regulates ETH biosynthesis and is stimulated by osmotic stress in tomato fruit (Fig. 7). This observation suggests that SnRK2‐I may be conservatively involved in osmotic stress‐triggered ETH biosynthesis in climacteric fruits. However, whether this mechanism is conserved in other plants remains unclear. Additionally, SnRK2s are reported to be functionally redundant (Fujii *et al*., 2011; Lin *et al*., 2020). However, we established that MdSnRK2‐I and SlSnRK2‐I regulate ETH biosynthesis synergistically rather than redundantly. Whether SnRK2‐II and SnRK2‐III are involved in regulating ETH biosynthesis requires further study.

ETH controls the ripening of climacteric fruits (Chang, 2016; Park *et al*., 2021). Tomato fruit are a common model in ETH‐mediated ripening studies (Li *et al*., 2021; Park *et al*., 2021). Recently, our understanding of the transcriptional regulation of ETH‐mediated fruit ripening has improved (Barry *et al*., 2000; Alexander & Grierson, 2002; Cara *et al*., 2008; Gapper *et al*., 2013; Kou *et al*., 2016; Li *et al*., 2017). SlHB1 (LeHB‐1) is an important regulator of tomato fruit ripening and *ACO1*‐mediated ETH biosynthesis (Lin *et al*., 2008). MdHB1, which is homologous to SlHB1 (LeHB‐1), is a regulator of anthocyanin accumulation in apple fruit (Jiang *et al*., 2017). Our results showed that SlHB1 (LeHB‐1) has two homologs in apple, MdHB1 and MdHB2 (Fig. S4a,b). These transcription factors increased *MdACO1* expression and therefore regulated ETH biosynthesis (Figs 5, S4e). Further analysis showed that MdHB1 and MdHB2 could be phosphorylated by MdSnRK2‐I, which enhanced their effects on *MdACO1* by improving their protein stability and transcriptional activity (Figs 4, 5). These results reveal the post‐transcriptional regulatory mechanism of HB transcription factors in ETH‐mediated fruit ripening. Additionally, MdSnRK2‐I was found to regulate MdACS1 degradation (Fig. 2b,c), suggesting that MdSnRK2‐I can act as a molecular switch of these two rate‐limiting steps to control ETH biosynthesis efficiently. However, whether SnRK2‐I protein kinases fully control ETH biosynthesis will need to be verified in fruit whose genomes have been edited to have altered MdSnRK2‐I.

We also established that SlSnRK2‐I regulates tomato fruit ripening by affecting ETH biosynthesis (Fig. 7a,b). Fruit ripening is a complex process including the accumulation of pigment, sugar, acid, aroma compounds and other substances (Adams‐Phillips *et al*., 2004; Gapper *et al*., 2013; Li *et al*., 2021). Both ETH and osmotic stress affect fruit quality (Alexander & Grierson, 2002; Cara & Giovannoni, 2008; Li *et al*., 2021). Drought and salinity significantly reduce the yield and quality of apple fruit (Dong *et al*., 2011; Ma *et al*., 2017a,b). However, moderate drought can improve apple fruit quality (Ma *et al*., 2017a,b). Future studies should focus on identifying the SnRK2‐I substrates associated with fruit quality and clarifying the underlying role of SnRK2‐I in osmoregulation and ripening. This information could help to identify candidate genes/phosphorylation sites and thereby improve our understanding of the balance between stress responses and fruit quality (Zhang *et al*., 2011; Lin *et al*., 2020; Maszkowska *et al*., 2021). FaSnRK2.6, a member of the SnRK2‐III family, is a negative regulator of strawberry fruit ripening and anthocyanin accumulation (Han *et al*., 2015). Therefore, the role of other SnRK2s in ripening of climacteric and nonclimacteric fruits should be explored to fully understand post‐transcriptional regulation of ripening.

## Author contributions

BL and WJ designed the study and conceived the experiments; WJ and SZ drafted the manuscript; MJ performed the experiments and analyzed the data; XL, WW, TL, ZD, YC, KZ, HZ, WM, QF, LL and JY assisted with some of the experiments; and BL and MJ wrote the manuscript.

## Supporting information


**Fig. S1** Ethylene production during fruit development and identification of *SnRK2* genes expressed in apple fruit.
**Fig. S2** Phosphorylation peptides of MdSnRK2.4/2.9 in apple fruit flesh at 85 DPA and 105 DPA.
**Fig. S3** Detection of MdSnRK2 antibody specificity and gene expression related to ETH synthesis.
**Fig. S4** Screening of MdHBs implicated in ETH production in fruits.
**Fig. S5** SnRK2 subfamily 1 protein kinases regulate tomato fruit ripening by mediating ETH biosynthesis and signaling.

**Table S1** Phosphorylation peptides of MdSnRK2.4/2.9 in apple fruit flesh at 85 DPA and 105 DPA.
**Table S2** IP‐MS analysis of MdSnRK2.9‐interacting proteins.

**Table S3** List of genes mentioned in this study.
**Table S4** Primers used in this study.
Please note: Wiley Blackwell are not responsible for the content or functionality of any Supporting Information supplied by the authors. Any queries (other than missing material) should be directed to the *New Phytologist* Central Office.

## Data Availability

The data that support the findings of this study are available on request from the corresponding author.
