# Supplementary material for: SnRK2 subfamily I protein kinases regulate ethylene biosynthesis by phosphorylating HB transcription factors to induce ACO1 expression in apple
Source: New Phytol. 2022 Mar 22;234(4):1262–77. doi: 10.1111/nph.18040 (PMC9314909; doi:10.1111/nph.18040)
Supplement: Supplementary file 1 — Fig. S1 Ethylene production during fruit development and identification of SnRK2 genes expressed in apple fruit. Fig. S2 Phosphorylation peptides of MdSnRK2.4/2.9 in apple fruit flesh at 85 DPA and 105 DPA. Fig. S3 Detection of MdSnRK2 antibody specificity and gene expression related to ETH synthesis. Fig. S4 Screening of MdHBs implicated in ETH production in fruits. Fig. S5 SnRK2 subfamily 1 protein kinases regulate tomato fruit ripening by mediating ETH biosynthesis and signaling. [file NPH-234-1262-s003.pdf]

New Phytologist

***New Phytologist* Supporting Information**

Article title: SnRK2 subfamily I protein kinases regulate ethylene biosynthesis by phosphorylating HB transcription factors to induce *ACO1* expression in apple

Authors: Meiru Jia, Xingliang Li, Wei Wang, Tianyu Li, Zhengrong Dai, Yating Chen, Kaikai Zhang, Haocheng Zhu, Wenwen Mao, Qianqian Feng, Liping Liu, Jiaqi Yan, Silin Zhong, Bingbing Li*, and Wensuo Jia*

Article acceptance date: 09 February 2022

The following Supporting Information is available for this article:

**Figure S1**. Ethylene production during fruit development and identification of SnRK2 genes expressed in apple fruit.

**Figure S2.** Phosphorylation peptides of MdSnRK2.4/2.9 in apple fruit flesh at 85 d post-anthesis (DPA) and 105 DPA.

**Figure S3.** Detection of MdSnRK2 antibody specificity and gene expression related to ethylene synthesis.

**Figure S4.** Screening of MdHBs implicated in ethylene production in fruits.

**Figure S5**. SnRK2 subfamily 1 protein kinases regulate tomato fruit ripening by mediating ethylene biosynthesis and signaling.

**Table S1.** List of genes mentioned in this study.

**Table S2.** Primers used in this study.

**Table S3.** Phosphorylation peptides of MdSnRK2.4/2.9 in apple fruit flesh at 85 d post-anthesis (DPA) and 105 DPA.

**Table S4.** IP-MS analysis of MdSnRK2.9-interacting proteins

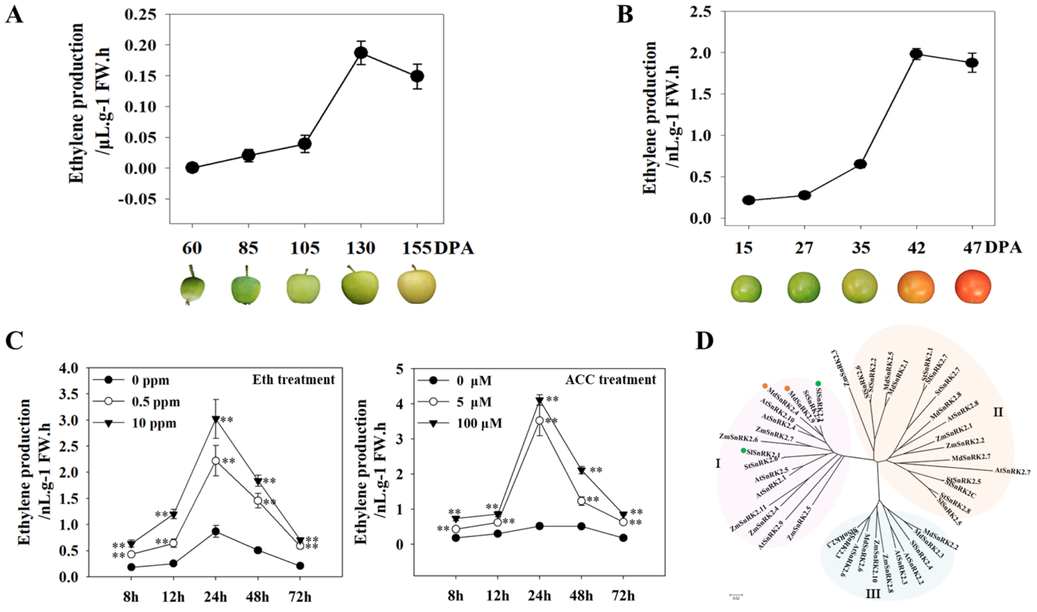


**Figure S1. Ethylene production during fruit development and identification of SnRK2 genes expressed in apple fruit. Related to Figure 1.**

(A) Ethylene production during 'Golden Delicious' apple fruit development and ripening. Fruit were collected at 60, 85, 105, 130 and 155 d post-anthesis (DPA). Values are means ± SD of three biological replicates.

(B) Ethylene production during 'Micro Tom' tomato fruit development and ripening. Fruit were collected at 15, 27, 35, 42 and 47 DPA. Values are means ± SD of three biological replicates.

(C) Effect of ethylene (Eth; left) and ACC (right) treatment on ethylene production in apple callus cells. Labels below bars denote the corresponding time after treatment. Values are means ± SD of three biological replicates. The asterisks indicate significant difference compared with the 0 ppm ethylene treatment control (**$p$ < 0.01, Student's $t$-test).

(D) Phylogenetic tree of the SnRK2 protein family from *Arabidopsis thaliana* (At), *Malus domestica* (Md), *Solanum lycopersicum* (Sl), *Zea mays* (Zm), and *Solanum tuberosum* (St), indicating that MdSnRK2.4 and MdSnRK2.9 as well as SlSnRK2.1 and SlSnRK2.2 were categorized into one subclass. The green circles given in this panel indicate SlSnRK2.1 and SlSnRK2.2, the orange circles indicate MdSnRK2.4

and MdSnRK2.9.

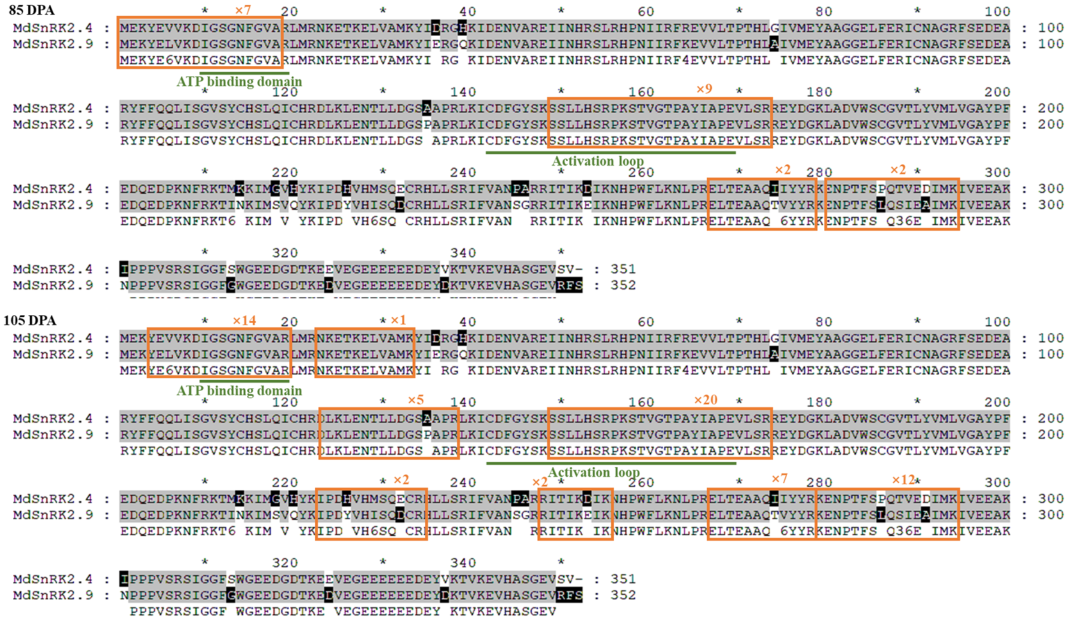


**Figure S2. Phosphorylation peptides of MdSnRK2.4/2.9 in apple fruit flesh at 85 d post-anthesis (DPA) and 105 DPA. Related to Figure 1.**

Total proteins were extracted from apple fruit flesh at 85 and 105 DPA, and then anti-MdSnRK2.4/2.9 specific antibodies were used to immunoprecipitate MdSnRK2.4/2.9 proteins. Phosphorylation levels of MdSnRK2.4/2.9 proteins were analyzed by LC-MS/MS. The ATP-binding domain and the activation loop are underlined in green.

Recognized phosphorylation peptides are marked with orange boxes, and the numbers above the boxes indicate the number of times a peptide was identified.

A

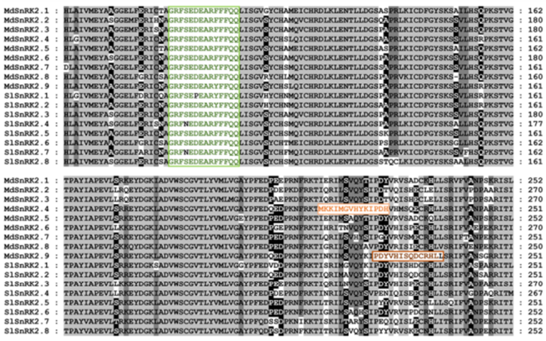

C

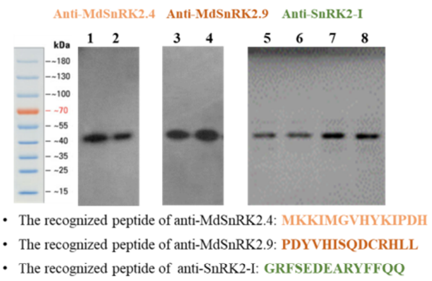


B

| Gene | kD | Gene | kD |
|---|---|---|---|
| MdSnRK2.1 | 38.4 | SlSnRK2.1 | 41.0 |
| MdSnRK2.2 | 40.8 | SlSnRK2.2 | 41.6 |
| MdSnRK2.3 | 40.4 | SlSnRK2.3 | 41.1 |
| MdSnRK2.4 | 40.2 | SlSnRK2.4 | 40.2 |
| MdSnRK2.5 | 38.4 | SlSnRK2.5 | 39.3 |
| MdSnRK2.6 | 40.9 | SlSnRK2.6 | 38.4 |
| MdSnRK2.7 | 38.4 | SlSnRK2.7 | 37.9 |
| MdSnRK2.8 | 38.6 | SlSnRK2.8 | 39.3 |
| MdSnRK2.9 | 40.4 | | |

**Figure S3. Detection of MdSnRK2 antibody specificity and gene expression related to ethylene synthesis. Related to Figure 3.**

(A) Alignment of the protein sequences of SlSnRK2.1-8 and MdSnRK2.1-9. Sequences were aligned using ClustalX 2.0.12 with default settings. Alignments were edited and marked using GeneDoc. Recognized peptides of anti-MdSnRK2.4, anti-MdSnRK2.9, and anti-SnRK2-I are marked with boxes corresponding to the colors in (C).

(B) Predicted molecular weight of SlSnRK2.1-8 and MdSnRK2.1-9 proteins. Predictions were made using DNAMAN.

(C) Specific detection of SnRK2 antibody. 1, 3, and 7 denote apple fruit flesh; 2, 4, and 8 denote apple fruit calli; and 5 and 6 denote tomato fruit.

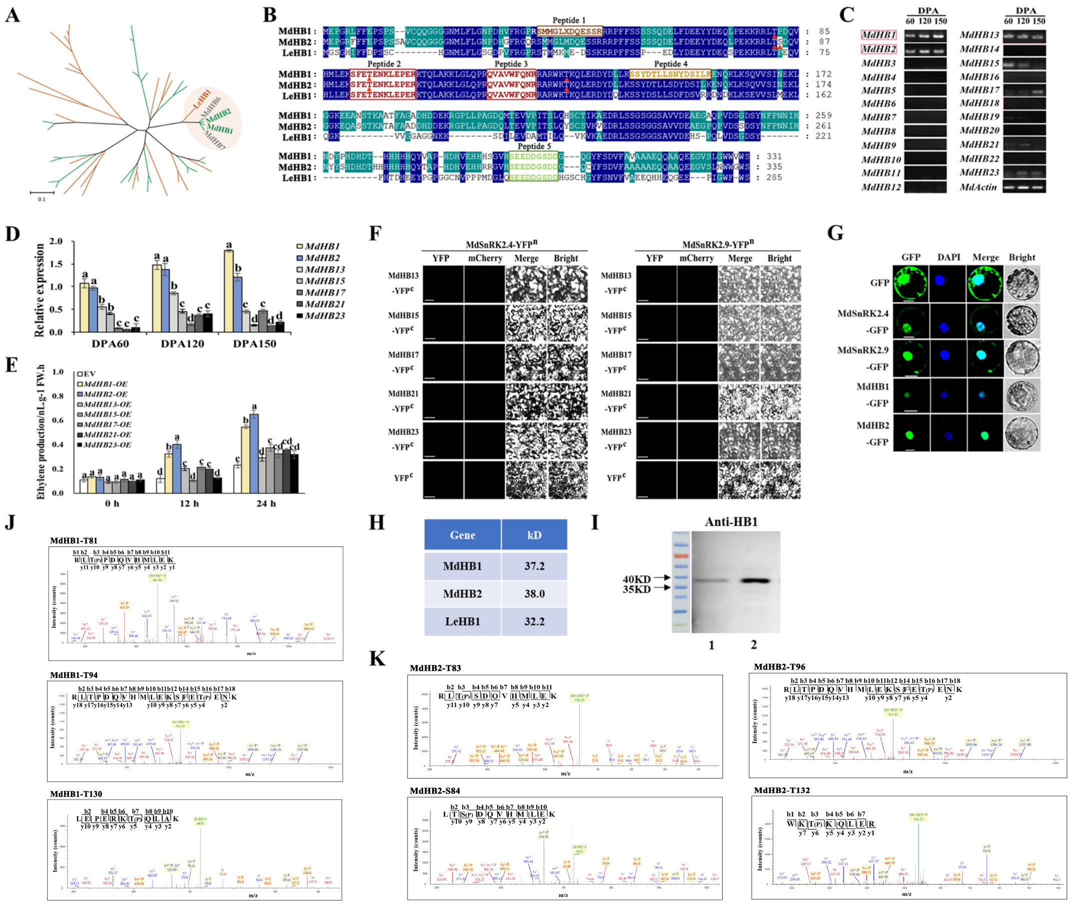


| Gene | kD |
|---|---|
| MdHB1 | 37.2 |
| MdHB2 | 38.0 |
| LeHB1 | 32.2 |

**Figure S4. Screening of MdHBs implicated in ethylene production in fruits. Related to Figure 4.**

(A) Phylogenetic tree showing that MdHB1, MdHB2, and LeHB1 are categorized into the homeobox transcription factor subfamily. The green branches given in this panel indicate MdHBs and orange branches indicate LeHBs.

(B) Alignment of the protein sequences of MdHB1, MdHB2 and LeHB1. Peptides 1-4, the MdSnRK2.9 interacting protein (ID V5LLY3) containing peptides by IP-MS. Peptide 5, the peptide for antibody production. Solid red triangle, the phosphorylation sites of MdHB1 and MdHB2 mediated by MdSnRK2.4 and MdSnRK2.9.

(C and D) Expression of different *MdHBs* in apple fruit flesh at different developmental stages. The two red boxes in (C) indicate *MdHB1* and *MdHB2*. Bars in (D) represent means ± SD of three biological replicates, and different letters indicate significant differences among the genes and the empty vector control (Tukey's HSD test; $p < 0.01$).

(E) Screening of *MdHB1*, *MdHB2*, *MdHB13*, *MdHB15*, *MdHB17*, *MdHB21*, and *MdHB23* for their potential individual roles in ethylene biosynthesis regulation. Apple callus cells were transformed with an *Agrobacterium tumefaciens* strain containing vectors harboring the seven *MdHB* genes driven by the *CaMV 35S* promoter. Apple callus cells were examined for ethylene production at different times after transformation. Bars represent means ± SD of three biological replicates. Different letters indicate significant differences among the genes and the empty vector control (Tukey's HSD test; $p < 0.01$).

(F) Screening for MdHB proteins that interact with MdSnRK2.4 and MdSnRK2.9 using a BiFC protocol. MdHBs and MdSnRK2.4/2.9 were fused with the C and N terminus of yellow fluorescent protein (YFP), designated as YFPc and YFPn, respectively. Different combinations of the fused constructs were co-transformed into tobacco (*Nicotiana tabacum*) cells, and the cells were visualized using confocal microscopy. YFP and bright field were excited at 488 and 543 nm, respectively. Bars = 20 μm.

(G) Cellular localization analysis of MdSnRK2.4 and MdSnRK2.9 in maize protoplasts transiently expressing GFP-fused MdSnRK2.4 and MdSnRK2.9, as examined by confocal laser-scanning microscopy. Scale bars, 20 μm.

(H) Specific detection of HB1 antibody1, apple fruit flesh; 2, tomato fruit.

(I) Predicted molecular weights of MdHB1, MdHB2, and LeHB1. Weights were predicted with DNAMAN.

(J and K) Identification of the phosphorylated sites of MdHB1 (J) and MdHB2 (K) using LC-MS/MS.

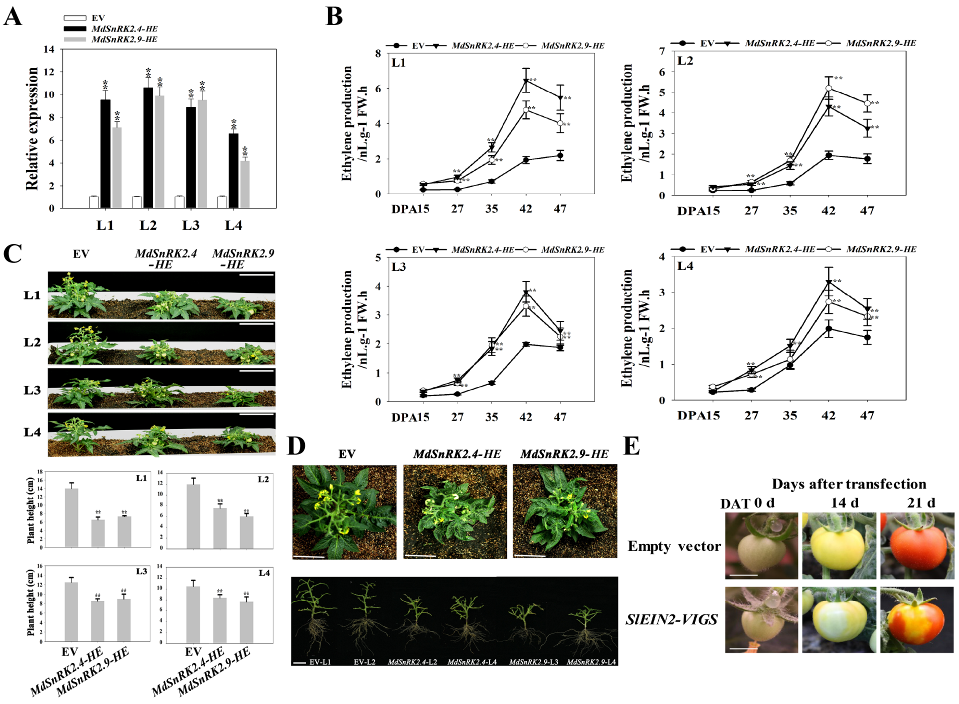


**Figure S5. SnRK2 subfamily 1 protein kinases regulate tomato fruit ripening by mediating ethylene biosynthesis and signaling. Related to Figure 6 and 7.**

(A) Relative *MdSnRK2.4/2.9* gene expression in transgenic plants as determined by qRT-PCR. Values are means ± SD, $n$ = 10. The asterisks indicate significant difference compared with the EV control (**$p < 0.01$, Student's t-test). L, line.

(B) Effect of heterologous *MdSnRK2.4* and *MdSnRK2.9* expression on ethylene production in tomato fruit. Tomato plants were stably transformed with *MdSnRK2.4* and *MdSnRK2.9*. Ethylene production was determined at different developmental stages. Values are means ± SD, $n$ = 4. The asterisks indicate significant difference compared with the EV control (**$p < 0.01$, Student's t-test).

(C) Plant height of *MdSnRK2.4* and *MdSnRK2.9* overexpression (OE) plants. Values are means ± SD, $n$ = 10. Asterisks denote significant differences between transgenic and empty vector (EV) plants (Student's *t*-test, **$p < 0.01$). Scale bar, 10 cm.

(D) Phenotypes of the *MdSnRK2.4* and *MdSnRK2.9* overexpression plants. Scale bar, 5 cm.

(E) The rate of fruit development in *SlEIN2-VIGS* (virus-induced gene silencing) plants and EV plants. Scale bar, 1 cm.
