# Supplementary material for: SnRK2 subfamily I protein kinases regulate ethylene biosynthesis by phosphorylating HB transcription factors to induce ACO1 expression in apple
Source: New Phytol. 2022 Mar 22;234(4):1262–77. doi: 10.1111/nph.18040 (PMC9314909; doi:10.1111/nph.18040)
Supplement: Supplementary file 2 — Table S1 Phosphorylation peptides of MdSnRK2.4/2.9 in apple fruit flesh at 85 DPA and 105 DPA. Table S2 IP‐MS analysis of MdSnRK2.9‐interacting proteins. [file NPH-234-1262-s002.pdf]

New Phytologist Supporting Information

Article title: SnRK2 subfamily I protein kinases regulate ethylene biosynthesis by phosphorylating HB transcription factors to induce *ACO1* expression in apple

Authors: Meiru Jia, Xingliang Li, Wei Wang, Tianyu Li, Zhengrong Dai, Yating Chen, Kaikai Zhang, Haocheng Zhu, Wenwen Mao, Qianqian Feng, Liping Liu, Jiaqi Yan, Silin Zhong, Bingbing Li*, and Wensuo Jia*

Article acceptance date: 09 February 2022

The following Supporting Information is available for this article:

Figure S1. Ethylene production during fruit development and identification of SnRK2 genes expressed in apple fruit.

Figure S2. Phosphorylation peptides of MdSnRK2.4/2.9 in apple fruit flesh at 85 d post-anthesis (DPA) and 105 DPA.

Figure S3. Detection of MdSnRK2 antibody specificity and gene expression related to ethylene synthesis.

Figure S4. Screening of MdHBs implicated in ethylene production in fruits.

Figure S5. SnRK2 subfamily 1 protein kinases regulate tomato fruit ripening by mediating ethylene biosynthesis and signaling.

Table S1. List of genes mentioned in this study.

Table S2. Primers used in this study.

Table S3. Phosphorylation peptides of MdSnRK2.4/2.9 in apple fruit flesh at 85 d post-anthesis (DPA) and 105 DPA.

Table S4. IP-MS analysis of MdSnRK2.9-interacting proteins.

Note: Figures S1-S5 and Table S3 are provided as separate files.

Table S1. List of genes mentioned in this study.

Gene name	Gene number	Gene name	Gene number
MdSnRK2.1	KJ563282	MdHB12	MDP0000119544
MdSnRK2.2	KJ563283	MdHB13	MDP0000509120
MdSnRK2.3	KJ563284	MdHB14	MDP0000564897
MdSnRK2.4	JX569851	MdHB15	MDP0000598064
MdSnRK2.5	KJ563285	MdHB16	MDP0000599845
MdSnRK2.6	KJ563286	MdHB17	MDP0000615948
MdSnRK2.7	KJ563287	MdHB18	MDP0000736852
MdSnRK2.8	KJ563288	MdHB19	MDP0000737128
MdSnRK2.9	KJ563289	MdHB20	MDP0000124827
MdHB1	MDP0000737672	MdHB21	MDP0000859459
MdHB2	MDP0000427227	MdHB22	MDP0000866008
MdHB3	MDP0000134241	MdHB23	MDP0000899816
MdHB4	MDP0000138651	SlSnRK2.1	AK329882
MdHB5	MDP0000150088	SlSnRK2.2	AK327274
MdHB6	MDP0000150465	LeHB1	Solyc02g086930.2.1
MdHB7	MDP0000152629	SlEIN2	AY566238
MdHB8	MDP0000206752	MdEIN2	MDP0000152033
MdHB9	MDP0000281698	MdACO1	AF030859
MdHB10	MDP0000311522	MdACS1	MDP0000370791
MdHB11	MDP0000316497		

Table S2. Primers used in this study.

Primer name	Primer sequence (5'-3')
**qRT-PCR**	
MdSnRK2.1-qRT-F	TTGAGAGAACAAACTATGCGTC
MdSnRK2.1-qRT-R	CCAGAAAAGTCACCACTGCA
MdSnRK2.2-qRT-F	AACCTTATATGTGATGCTGG
MdSnRK2.2-qRT-R	CTGCTGGTATAGTAGCCTCCT
MdSnRK2.3-qRT-F	GGAGAAATGTTTGAGCGAATAAG
MdSnRK2.3-qRT-R	GAGCTGGACTGCCATCCAAC
MdSnRK2.4-qRT-F	CTTACAGAAGCAGCTCAAATCA
MdSnRK2.4-qRT-R	GACGCTCACTTCTCCACTTGC
MdSnRK2.5-qRT-F	CTGCAGACTGCAAGCAACTA
MdSnRK2.5-qRT-R	GACCTCCTGCTTTGGATCCA
MdSnRK2.6-qRT-F	AGGGACAAGCAGACTGGTGA
MdSnRK2.6-qRT-R	GGATGCCTCAATGACCTGTG
MdSnRK2.7-qRT-F	GCTGGTGAAGGACAAATGGAGTG
MdSnRK2.7-qRT-R	GATCTCTGTGGCAAATTTGCATTG
MdSnRK2.8-qRT-F	AAGGCTGGCAAGAGACAAGCTC
MdSnRK2.8-qRT-R	GCAGGACCTCTTTGAATCGAAC

MdSnRK2.9-qRT-F	CTAAATCAACTGTGGGAACTCCT
MdSnRK2.9-qRT-R	TGGGATCTTGTACTGAACAGA
MdActin-qRT-F	AGGTCCATCCATTGTCCACAG
MdActin-qRT-R	TGCCAACCAAACTGACTTCAC
MdHB1-qRT-F	GACGAGGAATACTACGACGAGCAG
MdHB1-qRT-R	AGTAACACTGGCCGTCATCGCTAC
MdHB2-qRT-F	GACGAGGAATACTACGACGAGCAG
MdHB2-qRT-R	CCGCTGTGATGATGTTCTACATGA
MdHB3-qRT-F	CATCAGAGCGGTTATGGG
MdHB3-qRT-R	CGAATAGCAACCGACGTG
MdHB4-qRT-F	ACGCGAATCTGCAAACCCTAAA
MdHB4-qRT-R	GCTTTGAGGGCGCTGTAGTCTC
MdHB5-qRT-F	GGGTCAACGGCGGTCGAAAT
MdHB5-qRT-R	ACACCTGCATCCACGCCTCT
MdHB6-qRT-F	CTGTTCGACGAGGAATGCTACGAC
MdHB6-qRT-R	GGAGAAGTAACACTGGCCGTCATC
MdHB7-qRT-F	GACGAGGAATACTACGACGAGCAG
MdHB7-qRT-R	CGTAAGCTTGTTGCGGATAACCTT
MdHB8-qRT-F	TACGGTTCTTCTGCGGACATGA
MdHB8-qRT-R	GGCAAGAGGGTCTTTCCGAGAT
MdHB9-qRT-F	CGGCTCACGGCGAATCAAGT
MdHB9-qRT-R	GGAATCCGCCGGCTCTAACA
MdHB10-qRT-F	ACCGGGAGCTGCTACTACAA
MdHB10-qRT-R	TCCCACCACTGATAATCACC
MdHB11-qRT-F	TGCAGTTGGCCAAAGAGCTAGG
MdHB11-qRT-R	GCTGAAAGGGCGAGAGAACGAG
MdHB12-qRT-F	TGAGACATCATCGCAAGG
MdHB12-qRT-R	CGAATAGAAACCGACGTG
MdHB13-qRT-F	GTGCTGCAAGCCAACTACAATA
MdHB13-qRT-R	AAGCTTTGGGAAGATGTATGGA
MdHB14-qRT-F	GCAAGAAGATAACGCAGCGAGCAA
MdHB14-qRT-R	TGGAACGGAAAGCAATTCATCGAC
MdHB15-qRT-F	GGCGTGGCATCGTTTCTAGGAA
MdHB15-qRT-R	GATTGCCGGTGTTCTCGAAATG
MdHB16-qRT-F	GAGGGTCTACGGCGGCGGAAGT
MdHB16-qRT-R	TCGTGCTCAAACTGGCGGAACA
MdHB17-qRT-F	GAAGAACAAGGGCACAAACACGAG
MdHB17-qRT-R	TCCGCTCCAATTCCTCTTCTAGTCT
MdHB18-qRT-F	GGGCGGTGTCAAAGATGTTAGCAG
MdHB18-qRT-R	TTGAGTGAAGTGGAAGCCGGAGAC
MdHB19-qRT-F	ATTTGTCGGACGACGGAT
MdHB19-qRT-R	TGGAGGAGTTGGGTTGTG3
MdHB20-qRT-F	AAATGCCACCATCACGAC

MdHB20-qRT-R	GGGTTGGGTTATGCTTGC
MdHB21-qRT-F	CAAGGAGTTGCAGGCAATGTTAGA
MdHB21-qRT-R	GAACGCCATTTGAATCACTATCTG
MdHB22-qRT-F	TGATTCTTTGTTCCTTTCTCGGTC
MdHB22-qRT-R	ACCACCTCCCTGCATCTGACTCGC
MdHB23-qRT-F	CATAGAGCCACAGTCTGCGATGA
MdHB23-qRT-R	ACTCGATCCATCTGCCGTCTGATT
MdACO1-qRT-F	TCAAGGATGGTGAATGGGTG
MdACO1-qRT-R	AATGAGTCGTTGCCTGGGTT
MdACO2-qRT-F	GCTGTCCAGTCCGAAATCCA
MdACO2-qRT-R	GGGACATGGAGGGTAGTTGC
MdACO3-qRT-F	GACACAGTGGAGAGGCTGAC
MdACO3-qRT-R	CACACAACAAGTCCAGCAGC
MdACS1-qRT-F	CCAAGAAGCCGAAAAACGCA
MdACS1-qRT-R	GGTGGATGCCCTTGTCTTCA
MdACS3A-qRT-F	TCTGCCTTCTCATCCTCCGA
MdACS3A-qRT-R	CACAGTGCCAACTCGAAAGC
MdACS5A-qRT-F	CCGGAGCTCATGAGACGATC
MdACS5A-qRT-R	CCACCCCAAATCTCGGTCAA
MdEIN2-qRT-F	TGGAGGGATCCAGATCTGCA
MdEIN2-qRT-R	CTACTTCCTCCATCGTCGCC
SlACO1-qRT-F	ACAAACAGACGGGACACGAA
SlACO1-qRT-R	CTCTTTGGCTTGAAACTTGA
SlEIN2-qRT-F	CTTTCTGCTTCTGTGCCAA
SlEIN2-qRT-R	CTTGCCCAGCCAATACAT
SlSnRK2.1-qRT-F	CAGTGAACCCGAAGCTCGAT
SlSnRK2.1-qRT-R	CCGGTGCAATATAGGCTGGT
SlSnRK2.2-qRT-F	AGGGAGTTGACAGAAGCAGC
SlSnRK2.2-qRT-R	TTCGTCGTCATCCTCCTCCT
SlActin-qRT-F	TCATGTTTGAGACCTTCAACGT
SlActin-qRT-R	CGGTGATTTCCTTGCTCATACG
**Vector construction**	
pCambia1304-MdSnRK2.4-F	TCTAGAATGGAAAAGTACGAGGTTGTCA
pCambia1304-MdSnRK2.4-R	GGTACCTCAGCTGACGCTCACTTCTC
pCambia1304-MdSnRK2.9-F	GGTACCATGGAGAAGTACGAGCTTGTTAAG
pCambia1304-MdSnRK2.9-R	GAATTCTCAGCTGAAGCGCACTTCTCCAC
pCambia1304-SlSnRK2.1-F	GGCGCGCCATGGAGAAATACGAGCTTGTGAAG
pCambia1304-SlSnRK2.1-R	ACTAGTGGTGAGACGAACTTCCCCTAAG
pCambia1304-SlSnRK2.2-F	GGCGCGCCATGCAGAATTACGAAGTTGTGAAGG
pCambia1304-SlSnRK2.2-R	ACTAGTAGCATCATCATGTATGATATGGAACTC
TRV2-SlSnRK2.1-F	GAATTCTTGTTGCCATGAAATACATTGAGC
TRV2-SlSnRK2.1-R	GGTACCATCATATTCACGGCGCGATAGAAC
TRV2-SlSnRK2.2-F	GAATTCGGCATCCAAACATAATTCGCTTCA

TRV2-SlSnRK2.2-R	GGTACCTCAGCCAGCTTGCCATCATATTCC
TRV2-SlEIN2-F	TAAGGTTACCGAATTCTGGAAATGTCCCTGTAGGCC
TRV2-SlEIN2-R	AGACGCGTGAGCTCGGTACCCCCATCATCTTGCCTAAAT
pCambia1301:MdHB1-F	ATGGAGCCGGGCCGTTTTTTCTTTG
pCambia1301:MdHB1-R	TTATGACCATCCCCACCAGTTCAAAG
pCambia1301:MdHB2-F	ATGGAGCCGGGCCGTCTTTT
pCambia1301:MdHB2-R	CTAGGACCACACCCACCACC
SPYCE-MdEIN2-F	ATGGAATCCACCAATCCTAATGCT
SPYCE-MdEIN2-R	CCCATAAGGAGCAGATGTCAGA
35S-MdSnRK2.4-F	GAATTCAAGGTAGTTCCCACTGAATCAAAGGCCAT
35S-MdSnRK2.4-R	CTCGAGTCAGCTGACGCTCACTTCTCCACTTG
35S-MdSnRK2.9-F	GAATTCAAGGTAGTTCCCACTGAATCAAAGGCCAT
35S-MdSnRK2.9-R	CTCGAGTCAGCTGAAGCGCACTTCTCCACTTG
Pro-ACO1-F	GTCGACCTATAGAGACCGATTTTATTATGTG
Pro-ACO1-R	AGATCTTTCTCTTTGGATTGCTTGGTTTG
**Protein interaction assay**	
**Yeast two-hybrid assay**	
GBD-MdSnRK2.4-F	GAATTCATGGAAAAGTACGAGGTTG
GBD-MdSnRK2.4-R	GGATCCGCTGACGCTCACTTCTC
GAD-MdHB2-F	GAATTCATGGAGCCGGGCCGTCTTTTC
GAD-MdHB2-R	GGATCCGGACCACACCCACCACC
GBD-MdSnRK2.4-STKC-F	GAATTCTACGAGGTTGTCAAGGATATTG
GBD-MdSnRK2.4-STKC-R	GGATCCTCAATATGCTGGAGTTCCCACTG
GBD-MdSnRK2.4-UR-F	GAATTCATTGCTCCTGAGGTTCTTTCTC
GBD-MdSnRK2.4-UR-R	GGATCCTCAGCTGACGCTCACTTCTC
GBD-MdSnRK2.9-F	GAATTCATGGAGAAGTACGAGCTTGTTAAG
GBD-MdSnRK2.9-R	GGATCCGCTGAAGCGCACTTCTCCAC
GBD-MdSnRK2.9-STKC-F	GAATTCTACGAGCTTGTTAAGGATATTGGATC
GBD-MdSnRK2.9-STKC-R	GGATCCATATATGCAGGAGTTCCCACAG
GBD-MdSnRK2.9-UR-F	GAATTCATTGCACCTGAAGTTCTTTCTC
GBD-MdSnRK2.9-UR-R	GGATCCGCTGAAGCGCACTTCTCCAC
GAD-MdHB2-UR-F	GAATTCATGGAGCCGGGCCGTCTTTTC
GAD-MdHB2-UR-R	GGATCCCTACTGCTCGTCGTAGTATTCCTC
GAD-MdHB2-HOX-F	GAATTCCTGCCGGAGAAGAAGCGCCG
GAD-MdHB2-HOX-R	GGATCCCTACTCGAGCTGCTTGGTCTTC
GAD-MdHB2-HALZ-F	GAATTCCGGGACTACGACCTTCTCAA
GAD-MdHB2-HALZ-R	GGATCCCTAGGACCACACCCACCACC
GAD-MdHB1-F	GAATTCATGGAGCCGGGCCGTTTTTTCTTTG
GAD-MdHB1-R	GGATCCCCATCCCCACCAGTTCAAAGAAAC
GAD-MdHB1-UR-F	GAATTCATGGAGCCGGGCCGTTTTTTCTTTG
GAD-MdHB1-UR-R	GGATCCTAGTATTCCTCGTCGAACAGTTC
GAD-MdHB1-HOX-F	GAATTCCTGCCGGAGAAGAAGCGCCG
GAD-MdHB1-HOX-R	GGATCCCGAGCTGCTTGGTCTTCCACCGA

GAD-MdHB1-HALZ-F	GAATTCCGCGATTACGACCTTCTCAA
GAD-MdHB1-HALZ-R	GGATCCCCATCCCCACCAGTTCAAAGAAAC
**BiFC**	
SPYCE-MdHB1-F	ATGGAGCCGGGCCGTTTTTCTTTG
SPYCE-MdHB1-R	TGACCATCCCCACCAGTTCAAAGAAAC
SPYCE-MdHB2-F	ATGGAGCCGGGCCGTCTTTTC
SPYCE-MdHB2-R	GGACCACACCCACCACC
pMDC83-MdSnRK2.4-GFP-F	TTAATTAAATGGAAAAGTACGAGGTTGTCA
pMDC83-MdSnRK2.4-GFP-R	GGCGCGCCAGCTGACGCTCACTTCTC
pMDC83-MdSnRK2.9-GFP-F	TTAATTAAATGGAGAAGTACGAGCTTGTTAAG
pMDC83-MdSnRK2.9-GFP-R	GGCGCGCCAGCTGAAGCGCACTTCTCCAC
pMDC83-MdHB1-GFP-F	TTAATTAAATGGAGCCGGGCCGTTTTTCTTTG
pMDC83-MdHB1-GFP-R	GGCGCGCCTATGACCATCCCCACCAGTTCAAAGAAAC
pMDC83-MdHB2-GFP-F	TTAATTAAATGGAGCCGGGCCGTCTTTTC
pMDC83-MdHB2-GFP-R	GGCGCGCCGGACCACACCCACCACC
pMDC83-SlSnRK2.1-GFP-F	TTAATTAAATGCAGAATTACGAAGTTGTGAAG
pMDC83-SlSnRK2.1-GFP-R	GGCGCGCCAGCATCATCATGTATGATATGGA
pMDC83-SlSnRK2.2-GFP-F	TTAATTAAATGGAGAAATACGAGCTTGTGA
pMDC83-SlSnRK2.2-GFP-R	GGCGCGCCGGTGAGACGAACTTCC
SPYCE-MdHB13-YFPc-F	ATGGCCGGCGGGAGGGTCAACG
SPYCE-MdHB13-YFPc-R	GTAAGACCAGGACCAAAAGGCATGATC
SPYCE-MdHB15-YFPc-F	ATGACTACCAATGGGATGGTCTTCTTCTC
SPYCE-MdHB15-YFPc-R	ATTGAATTGATGTTGTTCCAACCAAGG
SPYCE-MdHB17-YFPc-F	ATGGAAAGGAGGAAAGAGGAGTGCTATG
SPYCE-MdHB17-YFPc-R	AGCCCAGAAATTTAACCAGTGTAAATTAC
SPYCE-MdHB21-YFPc-F	ATGAAGAGGTTCAGCAGCTCAGATTCATTG
SPYCE-MdHB21-YFPc-R	CCAATGAAGACTTGGAGCTTGATCAACTG
SPYCE-MdHB23-YFPc-F	ATGGAGAGGAGTAAAGAGGAGTGCTATG
SPYCE-MdHB23-YFPc-R	GGTCCAGAAATTTAACCAGTGTGAATTA
SPYCE-MdEIN2-YFPc-F	ATGACCGGTTATGAATCATCCT
SPYCE-MdEIN2-YFPc-R	CCCATAAGGAGCAGATGTCAGA
SPYCE-MdACS1-YFPc-F	ATGCGCATGTTATCCAGAAACGCT
SPYCE-MdACS1-YFPc-R	TCTACCGGGAATAGGACCGCGGTC
**Co-IP**	
SPYNE-Flag-MdSnRK2.4-Myc-F	GGCGCGCCATGGAAAAGTACGAGGTTGTCAAGG
SPYNE-Flag-MdSnRK2.4-Myc-R	GGTACCGCTGACGCTCACTTCTCCACTTGCATG
SPYNE-Flag-MdSnRK2.9-Myc-F	GGCGCGCCATGGAGAAGTACGAGCTTGTTAAGG
SPYNE-Flag-MdSnRK2.9-Myc-R	GGTACCGCTGAAGCGCACTTCTCCACTTGCATG
**ChIP-PCR**	

E1-qRT-F	TGCTTTACCGTCTACAATTACCA
E1-qRT-R	TCATGTCATTTTCGATTATGAGCA
E2-qRT-F	CGTGAACGTGTGCATGAAGA
E2-qRT-R	GGGAACAGTAAGCACGTCCT
E3-qRT-F	AAGGGGAGGGGCTGAACTTA
E3-qRT-R	GCTAAGCTCACCCATCTCCC
E4-qRT-F	AAGGGGAGGGGCTGAACTTA
E4-qRT-R	TATAACCGGCATTACACGCC
E5-qRT-F	GTGTCACCAAGCCGTTCAAA
E5-qRT-R	TAAGTTCAGCCCCTCCCCTT
E6-qRT-F	CAGTGTCTAGCCGCCTAGTG
E6-qRT-R	CCATTTTTCCCAAGCATGCCA
E7-qRT-F	GTGAAAGTTGATGGTGGCCAC
E7-qRT-R	GCATTATGCGCCTCTTAAGATGT
**EMSA probe**	
EMSA-E1	TAATAGTGTATTATTTTGCTTTACCGTCTACAATTACCAAATTTGGAAAT
EMSA-E4	AAATAAGAAAAATTATGAATATTGTGTTCCAAACATCTTAAGAGGCGCA
EMSA-E6	CATCTCAGTCTTTTTCTCCACCCACCACC
**Prokaryotic expression**	
pEASY-E1-MdHB1-F	ATGGAGCCGGGCCGTTTTTTCTTTG
pEASY-E1-MdHB1-R	TATGACCATCCCCACCAGTTCAAAGAAAC
pEASY-E1-MdHB2-F	ATGGAGCCGGGCCGTTTTTTCTTTGAAC
pEASY-E1-MdHB2-R	TGACCATCCCCACCAGTTCAAAGAAACACC
pGEX4t-1-MdHB1-GST-F	GGATCCGAGCCGGGCCGTTTTTTCTTTG
pGEX4t-1-MdHB1-GST-R	GAATTCTATGACCATCCCCACCAGTTCAAAGAAAC
pGEX4t-1-MdHB2-GST-F	GGATCCGAGCCGGGCCGTTTTTTCTTTGAAC
pGEX4t-1-MdHB2-GST-R	GAATTCTGACCATCCCCACCAGTTCAAAGAAACACC
pGEX4t-1-MdSnRK2.4-GST-F	GGATCCGAAAAGTACGAGGTTGTCAAGGATATTG
pGEX4t-1-MdSnRK2.4-GST-2	GAATTCGCTGACGCTCACTTCTCCACTTGCATGTAC
pGEX4t-1-MdSnRK2.9-GST-F	GGATCCGAGAAGTACGAGCTTGTTAAGGATATTG
pGEX4t-1-MdSnRK2.9-GST-R	GAATTCGCTGAAGCGCACTTCTCCACTTGCATGTAC
pGEX4t-1-MdACS1-GST-F	CTGTTCCAGGGGCCCCTGGGATCCCGCATGTTATCCAGAAACGCT
pGEX4t-1-MdACS1-GST-R	CCGCTCGAGTCGACCCGGGAATTCTCATCTACCGGGAATAGGACCG
pGEX4t-1-MdACS1 mutant-GST-F	CTGTTCCAGGGGCCCCTGGGATCCCGCATGTTAGCCAGAAACGCT
pGEX4t-1-MdACS1 mutant-GST-R	ACCGGGAATAGGACCGCGGTCATCGAAGGATAGCCGGGAAACCCACTTTGTGAGCGCCTGTCTTCTTG
**Phosphorylation mutants**	
MdHB1-T81A-F	CGGAGAAGAAGCGCCGCCTCGCTCCTGACCA

MdHB1-T81A-R	CGAGGCGGCGCTTCTTCTCCGGCAGCTGCTC
MdHB1-T94A-F	GTACATATGCTGGAGAAGAGCTTTGAGGCAGAGAAC
MdHB1-T94A-R	GCCTCAAAGCTCTTCTCCAGCATATGTAC
MdHB1-T130A-F	CGCCGCGCTCGGTGGAAGGCCAAGCAGCTC
MdHB1-T130A-R	CCTTCCACCGAGCGCGGCGGTTCTGGAACCA
MdHB2-T83A/S84A-F	CGGAGAAGAAGCGCCGCCTC**G**CT**G**CTGACCAGGTG
MdHB2-T83A/S84A-R	**C**AG**C**GAGGCGGCGCTTCTTCTCCGGCAGCTG
MdHB2-T96A-F	CTGGAGAAGAGCTTTGAGGCTGAGAACAAGC
MdHB2-T96A-R	AGCCTCAAAGCTCTTCTCCAG
MdHB2-T132A-F	ACCGCCGCGCTCGGTGGAAG**G**CCAAGCAGCTC
MdHB2-T132A-R	**C**CTTCCACCGAGCGCGGCGGTTCTGGAACCAC

Table S4. IP-MS analysis of MdSnRK2.9-interacting proteins.

Accession	Description	Score	Coverage	Unique peptides	Peptides	PSMs
A0A076JYZ8	SnRK2.9 OS=Malus domestica PE=2 SV=1 - [A0A076JYZ8_MALDO]	794.4	78.41	26	33	373
A0A076K094	SnRK2.6 OS=Malus domestica PE=2 SV=1 - [A0A076K094_MALDO]	150.9	15.75	1	8	83
A0A1C7A0M2	Phi class glutathione S-transferase OS=Malus domestica GN=GSTF3 PE=2 SV=1 - [A0A1C7A0M2_MALDO]	147.44	62.91	10	10	81
P93270	Polygalacturonase-inhibiting protein OS=Malus domestica GN=mdpgip1 PE=2 SV=1 - [P93270_MALDO]	61.211	29.7	9	9	30
A3DSX0	Malate dehydrogenase OS=Malus domestica PE=2 SV=1 - [A3DSX0_MALDO]	33.961	25.3	7	7	16
V5LLY3	Homeobox-leucine zipper protein OS=Malus domestica PE=2 SV=1 - [V5LLY3_MALDO]	31.998	14.8	5	5	15
A0A0F7EVJ8	Ribulose bisphosphate carboxylase large chain (Fragment) OS=Malus domestica GN=rbcL PE=3 SV=1 - [A0A0F7EVJ8_MALDO]	23.903	13.59	1	5	13
Q9M6R1	High molecular weight heat shock protein OS=Malus domestica GN=Hsp2 PE=2 SV=1 -	23.355	14.46	8	8	14

	[Q9M6R1_MALDO]					
H9CU70	Ribulose bisphosphate carboxylase large chain (Fragment) OS=Malus platycarpa GN=rbcL PE=3 SV=1 - [H9CU70_MALPL]	20.472	12.53	1	5	10
G5ELM7	Actin (Fragment) OS=Malus domestica PE=2 SV=1 - [G5ELM7_MALDO]	17.733	18.73	5	5	9
A4IEB1	Pathogenesis-related protein 8 (Fragment) OS=Malus domestica GN=pr-8 PE=2 SV=1 - [A4IEB1_MALDO]	14.416	14.71	1	1	4
Q9ZRP9	Elongation factor 1-alpha OS=Malus domestica GN=eF-1alpha PE=2 SV=1 - [Q9ZRP9_MALDO]	14.254	11.19	1	5	9
Q2I2W1	Elongation factor 1 alpha (Fragment) OS=Malus domestica PE=4 SV=1 - [Q2I2W1_MALDO]	12.721	17.95	1	4	8
A0A0B5W3U1	Pectinesterase OS=Malus domestica PE=2 SV=1 - [A0A0B5W3U1_MALDO]	11.62	9.4	5	5	10
O65152	Putative cinnamyl alcohol dehydrogenase OS=Malus domestica GN=CAD PE=2 SV=1 - [O65152_MALDO]	11.435	12.92	3	3	8
S4ULE0	Lipoxygenase OS=Malus domestica GN=LOX1a PE=2 SV=1 - [S4ULE0_MALDO]	8.8724	7.07	5	5	6
P83336	Thaumatin-like protein 1b (Fragment) OS=Malus domestica PE=2 SV=1 - [TP1B_MALDO]	7.8355	11.32	2	2	4
Q5UFR1	14-3-3 family protein OS=Malus domestica PE=2 SV=1 - [Q5UFR1_MALDO]	7.4971	12.21	3	3	4
J7MPY1	ATP synthase subunit alpha OS=Malus domestica GN=atp1 PE=3 SV=1 - [J7MPY1_MALDO]	6.8316	6.86	3	3	4
Q9ZS53	Apgm protein OS=Malus domestica GN=apgm PE=2 SV=1 - [Q9ZS53_MALDO]	5.7677	8.94	3	3	4
Q8L6K9	Major allergen d 1 (Fragment) OS=Malus domestica GN=Mal d 1 PE=2 SV=1 - [Q8L6K9_MALDO]	4.5314	17.09	2	2	3
A0A1W5S4A5	Phenylpropene reductase 4	4.4929	8.77	2	2	2

	OS=Malus domestica GN=PhR4 PE=2 SV=1 - [A0A1W5S4A5_MALDO]					
S4U6N6	Lipoxygenase OS=Malus domestica GN=LOX7a PE=2 SV=1 - [S4U6N6_MALDO]	4.3763	4.61	3	3	3
H2FL90	Biphenyl synthase 3 OS=Malus domestica PE=2 SV=1 - [H2FL90_MALDO]	3.7882	10.82	3	3	6
A0A140C6X8	30S ribosomal protein S3 OS='Malus domestica' phytoplasma Madom GN=rpsC PE=3 SV=1 - [A0A140C6X8_9MOLU]	3.7605	3.23	1	1	2
C0LQ98	Monodehydroascorbate reductase OS=Malus domestica PE=2 SV=1 - [C0LQ98_MALDO]	3.6952	5.99	2	2	2
P93272	Elongation factor 1 alpha (Fragment) OS=Malus domestica GN=EF-1alpha PE=2 SV=1 - [P93272_MALDO]	3.0402	15.38	1	2	2
Q6V8M6	GTP-binding nuclear protein (Fragment) OS=Malus domestica PE=2 SV=1 - [Q6V8M6_MALDO]	2.6461	7.1	1	1	1
D6QSI2	Alcohol dehydrogenase (Fragment) OS=Malus domestica GN=Adh PE=4 SV=1 - [D6QSI2_MALDO]	1.8953	11.7	1	1	1
A5A4X2	Ascorbate peroxidase OS=Malus domestica GN=APX PE=2 SV=1 - [A5A4X2_MALDO]	1.7167	11.6	3	3	3
Q1KLZ1	L-3-cyanoalanine synthase 2, mitochondrial OS=Malus domestica GN=CAS2 PE=1 SV=1 - [CAS2_MALDO]	0	2.93	1	1	1
B7SKM8	Phosphoenolpyruvate carboxylase OS=Malus domestica PE=2 SV=1 - [B7SKM8_MALDO]	0	3.94	2	2	2
I6TQV4	Catalase 1 (Fragment) OS=Malus domestica GN=cat1 PE=4 SV=1 - [I6TQV4_MALDO]	0	6.49	1	1	2
G5CBV1	Receptor-like protein (Fragment) OS=Malus domestica PE=4 SV=1 - [G5CBV1_MALDO]	0	3.84	1	1	1
D9ZJ20	HD domain class transcription factor OS=Malus domestica GN=HD5 PE=2 SV=1 - [D9ZJ20_MALDO]	0	9.32	1	1	1

Q09K04	Caffeic acid O-methyltransferase OS=Malus domestica GN=omt1 PE=3 SV=1 - [Q09K04_MALDO]	0	5.75	1	1	1
A0A067XN06	WD40 domain-containing protein OS=Malus domestica GN=ATG18a PE=2 SV=1 - [A0A067XN06_MALDO]	0	3.29	1	1	1
